# Mood, food, and obesity

**DOI:** 10.3389/fpsyg.2014.00925

**Published:** 2014-09-01

**Authors:** Minati Singh

**Affiliations:** ^1^Department of Pediatrics, University of IowaIowa City, IA, USA; ^2^Department of Pediatrics, HHMI, University of IowaIowa City, IA, USA

**Keywords:** mood, depression, anxiety, food, obesity

## Abstract

Food is a potent natural reward and food intake is a complex process. Reward and gratification associated with food consumption leads to dopamine (DA) production, which in turn activates reward and pleasure centers in the brain. An individual will repeatedly eat a particular food to experience this positive feeling of gratification. This type of repetitive behavior of food intake leads to the activation of brain reward pathways that eventually overrides other signals of satiety and hunger. Thus, a gratification habit through a favorable food leads to overeating and morbid obesity. Overeating and obesity stems from many biological factors engaging both central and peripheral systems in a bi-directional manner involving mood and emotions. Emotional eating and altered mood can also lead to altered food choice and intake leading to overeating and obesity. Research findings from human and animal studies support a two-way link between three concepts, mood, food, and obesity. The focus of this article is to provide an overview of complex nature of food intake where various biological factors link mood, food intake, and brain signaling that engages both peripheral and central nervous system signaling pathways in a bi-directional manner in obesity.

## Introduction

**Figure 1:**
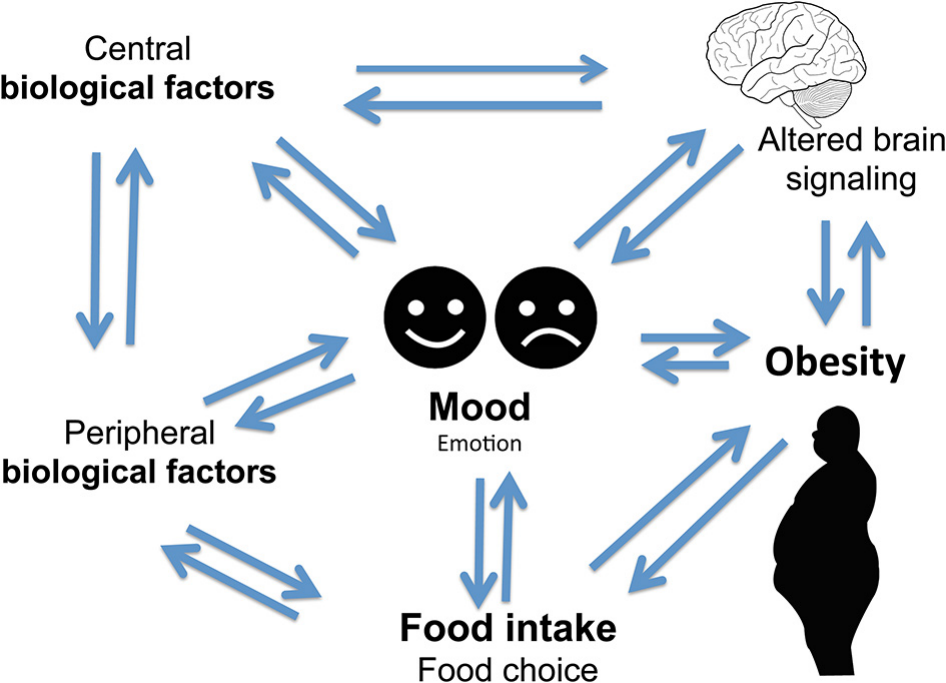
**Complex two-way relationship linking food intake, mood, and obesity**.

It is hypothesized that individuals engage in a variety of behaviors to regulate their mood (Morris and Reilly, 1987). Important among mood regulating behaviors is food consumption. The interaction between mood, emotional state, and feeding behaviors is complex and it is hypothesized that individuals regulate their emotions and mood by changing both food choices and quantities. It is also apparent that mood can affect the self-rewarding mechanisms of food consumption (Morris and Reilly, 1987). Specific types of food tend to be preferred under certain psychological conditions due to the influence of foods on the activity of brain reward centers (Figure 1) (Rangel, 2013; Jauch-Chara and Oltmanns, 2014; Weltens et al., 2014). Positive feedback loops can result in enhancement of appetite leading to obesity. Interestingly, highly palatable foods activate the same brain regions of reward and pleasure that are active in drug addiction (Volkow et al., 2012), suggesting a neuronal mechanism of food addiction leading to overeating and obesity (Davis et al., 2011, 2014; Dileone et al., 2012; Volkow et al., 2012; Dagher, 2013; Davis, 2013; Ziauddeen and Fletcher, 2013; Pai et al., 2014; Potenza, 2014). Dopamine, which directly activates reward and pleasure centers, affects both mood and food intake (Cantello et al., 1989; Diehl and Gershon, 1992; Fochtmann and Fink, 1992; Black et al., 2002; Cawley et al., 2013), further supporting the link between psychology and eating behaviors.

Mood disorders are often found in association with abnormal feeding behaviors. For example, depression and anxiety are comorbidities of obesity (Novick et al., 2005; Simon et al., 2006; Kloiber et al., 2007). Impairment in central nervous system (CNS) function has been linked to obesity that in turn impacts mental and physical health (Allison et al., 2009; Talen and Mann, 2009; Duarte et al., 2010). Obese individuals are at increased risk of developing depression (25, 26), and this risk is doubled in the presence of diabetes (Anderson et al., 2001; De Groot et al., 2001; Labad et al., 2010). Depressed mood is also associated with abdominal obesity and poor diet (Roberts et al., 2003; Dong et al., 2004; Simon et al., 2006; Luppino et al., 2010; Zhao et al., 2011; Hamer et al., 2012). A link between obesity and depression has been found in animal models of mood disorders (Lombard, 2000; Pawels and Volterrani, 2008; Dallman et al., 2003, 2005; Singh et al., 2007, 2009, 2011; Dallman, 2010; Chuang et al., 2011; Diz-Chaves, 2011; Maniam and Morris, 2012; Spence and Courbasson, 2012; Akubuiro et al., 2013; Kumar et al., 2013), suggesting that a common signaling pathway may underlie these phenotypes in both humans and animals.

There are numerous articles on the regulation of food intake, obesity, and mood. However, further exploration of the interaction among mood, food, and obesity is much needed. The aim of this review article is to highlight the complex interplay among mood, emotional state, and eating behaviors that influence body weight. This review provides an overview of known biological factors and foods that influence appetite and mood *via* brain signaling pathways. Specifically discussed are the foods and biological factors, which override the normal physiological requirements of appetite regulation, and how these factors influence in a bi-directional manner emotion, food, food intake, and obesity (Figure 1).

## Central nervous system in regulation of mood, food, and obesity

### Bi-directional link of food and emotion

In humans, eating behavior is complex and is affected by both mood and emotions (Lyman, 1982; Mehrabian, 1995; Macht, 1999; Macht and Simons, 2000). However, mood and emotions are distinct. Mood is characterized by psychological arousal in the absence of obvious stimuli that can last for several minutes or longer. In contrast, emotions are short-term affective response to reinforcing stimuli. Of all emotions, a study shows that frequent emotions such as, anger and joy have the strongest influence on appetite and food choice (Macht, 1999). Behavior based findings from human studies of questionnaires, field, and clinical studies suggest an integrative five way model that predicts five different aspects of emotional eating. These five aspects include: food choice, food intake, loss of cognitive controls, food modulating emotions, and emotion-congruent modulating eating, see review by Macht (2008). Therefore, depending on the state of negative emotions or distress, emotional eating is triggered where food intake can either increase or decrease within the same individuals (Ouwens et al., 2009). Emotional state has also been connected with addiction (Parylak et al., 2011). Sensory and psychological pathways influence food choice, the quantity, and meal frequency that may not be a part of normal physiological requirement. Many psychosomatic theories of obesity suggests that obese people overeat due to inability to perceive their physiological state, hunger, and satiety and that overeating reduce emotional discomfort and anxiety (Kaplan and Kaplan, 1957; Schachter, 1968; Bruch, 1985). The internal/external theory of obesity predicts that normal eaters alter their food intake to regulate their emotion, while obese people do not (Schachter, 1968; Canetti et al., 2002). Depending on whether an eater is restrained or emotional, stress and negative emotions could be associated with both increased and decreased motivation to eat; and under those circumstances, food choice differs (Herman and Mack, 1975). Thus, emotional distress influences emotional food choice and intake.

### Stress and food intake

There is a close interaction between food, mood, and stress (Benton and Donohoe, 1999; Oliver and Wardle, 1999; Gibson, 2006; Dallman, 2010; Bast and Berry, 2014). Stress can affect feeding behavior (Greeno and Wing, 1994; Yau and Potenza, 2013), resulting in either increased or reduced food intake depending on the types of external or psychological stressors (Oliver and Wardle, 1999; Gibson, 2006; Dallman, 2010; Yau and Potenza, 2013). Similarly, chronic stress can lead to either increased consumption of palatable and rewarding foods leading to obesity or a diminished appetite leading to weight loss (Cartwright et al., 2003; Adam and Epel, 2007; Tryon et al., 2013). Furthermore, following exposure to a stressor, studies show that intake of palatable foods reduce signs of stress and anxiety (Pecoraro et al., 2004; La Fleur et al., 2005; Maniam and Morris, 2010, 2012; Ulrich-Lai et al., 2010; Finger et al., 2011, 2012). Interestingly, stress-induced preference for palatable food is often seen in humans (Souquet and Rowland, 1989; Epel et al., 2004; Pecoraro et al., 2004; Christiansen et al., 2011; Gibson, 2012; Merali et al., 2013; Sharma et al., 2013; Sharma and Fulton, 2013; Meye and Adan, 2014; Park et al., 2014; Rho et al., 2014). Notably, this behavior is extended to animals (Dallman et al., 2003, 2005; Cottone et al., 2009). This suggests that a common neurobiological pathway maybe involved in food choice and patterns of eating behavior during stress.

### Mood and food intake

Mood states such as anxiety and depression affect food choice and energy metabolism. Overeating and obesity is often associated with depression and anxiety in humans which has also been reported in animal models (Novick et al., 2005; Simon and Von Korff, 2006; Kloiber et al., 2007; Singh et al., 2007, 2009; Akubuiro et al., 2013; Patterson and Abizaid, 2013; Sharma and Fulton, 2013). Both endocrine and metabolic conditions are exacerbated in major depression (Mcelroy et al., 2004; Simon et al., 2006; De Wit et al., 2010; Luppino et al., 2010; Marijnissen et al., 2011). Individuals experiencing depressed moods show preference for and consume palatable “comfort foods” as a mean to alleviate their negative feelings (Macht, 2008). Although on a short-term basis, palatable foods can provide some relief from negative emotions and mood states, chronic consumption of calorically-rich foods ultimately leads to obesity which in turn promotes vulnerability to depression and anxiety (Novick et al., 2005; Simon et al., 2006; Kloiber et al., 2007; Sharma and Fulton, 2013). Conversely, there are findings showing that prolonged high-fat feeding leads to negative emotional states, increased stress sensitivity, and altered basal corticosterone levels (Sharma et al., 2012). Thus, negative emotion impacts food choice and intake that in turns affects mood in a bi-directional manner.

Interestingly, other behaviors of reduced pleasure/reward experience, anxiety-like behavior, and heightened stress-induced hypothalamic pituitary adrenal axis (HPA) activation have been found in mice. Furthermore, after exposure to chronic high-fat diet and then switching to normal chow diet, mice showed craving for sucrose, high-fat foods, and displayed enhanced anxiety-like behavior (Sharma et al., 2012). Similar findings of increased behavioral and physiological signs of depression and anxiety have been reported in humans when switched from a high-fat sugar diet to regular diet (Avena et al., 2008; Teegarden and Bale, 2008; Cottone et al., 2009; Pickering et al., 2009; Iemolo et al., 2012; Sharma et al., 2012; Blasio et al., 2013). All together, these findings suggest that chronic high-fat feeding promotes negative emotional states and potentiates condition for enhanced sensitivity to stress that leads to continuous repetitive cycles of overeating, weight gain, and depressed mood.

### Food preference and mood

Hippocrates, father of modern medicine, said: “Let your food be your medicine, and your medicine be your food” (Prasad, 1998). Research from human trials and animal studies have shown that foods directly influence brain neurotransmitter systems which in turn has effects on mood and performance by altering the brain structure, chemistry, and physiology. Mood can also influence our food choices and expectations on the effects of certain foods can influence our sapiens. Some of those foods impacting mood are discussed below and summarized in Table 1 (Spring et al., 1982-1983; Rogers and Lloyd, 1994).

**Table 1:** **Summary of biological factors and food influencing mood, emotions, food intake, and brain signaling pathways**.

**Foods and biological factors**	**Influence on mood, emotion, food intake, and brain signaling pathways**	**References**
Chocolate	Increases pleasant feeling, reduce tension, and results in good mood via serotonin and cannabinoid receptors signaling	Ottley, 2000; Osman and Sobal, 2006; Parker et al., 2006b; Cartwright et al., 2007; Fletcher et al., 2007
Caffeine	Enhances alertness and increases anxiety and results in withdrawal symptoms in some individuals *via* cannabinoid CB1 receptor signaling pathway	Rogers, 1995; Acquas et al., 2002; Rossi et al., 2010
Omega-3 fatty acids	Influences neuroticism, mood, behavior, and plays a role in mood disorders. Omega-3 fatty acids in receptor functioning, neurotransmitters levels, and monoamine metabolism are all implicated in depression (see review Parker et al., 2006a)	Lombard, 2000; Young and Martin, 2003; Parker et al., 2006a; Van Strater and Bouvy, 2006; Conklin et al., 2007; Sanchez-Villegas et al., 2007; Stahl et al., 2008; Antypa et al., 2012; Moranis et al., 2012; Kang and Gleason, 2013; Grosso et al., 2014
Micronutrients	Thiamine plays a role in emotion, mood states, and cognitive functioning. The pathway is unknown	Benton et al., 1995, 1997; Benton and Donohoe, 1999
Iron	Iron deficiency results in depressed mood and lethargy. The pathway is unknown	Benton and Donohoe, 1999
Folic acid	Folic acid deficiency is associated with depressed mood. The pathway is unknown	Coppen and Bolander-Gouaille, 2005; Young, 2007
Ghrelin	Linked to stress mediated food reward behavior, depression, and anxiety via ghrelin receptor signaling pathway	Schanze et al., 2008; Barim et al., 2009; Kluge et al., 2009, 2011; Perello et al., 2010; Chuang et al., 2011; Diz-Chaves, 2011; Kumar et al., 2013
Serotonin	Linked to food intake, depression, and anxiety via serotonin receptor signaling pathway	Wurtman and Wurtman, 1989; Benton and Donohoe, 1999; Pepino et al., 2009; Shabbir et al., 2013
Dopamine	Linked to food reward behavior and mood via dopamine receptor signaling pathway	Cantello et al., 1989; Diehl and Gershon, 1992; Fochtmann and Fink, 1992; Berridge, 1996; Black et al., 2002; Davis et al., 2009; Cawley et al., 2013
Leptin	Linked to food intake, depression, anxiety, and mood disorder via leptin receptor signaling pathway	Collin et al., 2000; Asakawa et al., 2003; Lu et al., 2006; Finger et al., 2010; Liu et al., 2010; Sharma et al., 2010; Yamada et al., 2011; Guo et al., 2012, 2013
Adiponectin	Linked to depression and mood disorder. May involve adiponectin-induced inhibition of GSK-3β pathway	Arita et al., 1999; Maeda et al., 2001; Milan et al., 2002; Cnop et al., 2003; Delporte et al., 2004; Ryo et al., 2004; Leo et al., 2006; Narita et al., 2006; Hanley et al., 2007; Weber-Hamann et al., 2007; Ye et al., 2007; Yilmaz, 2008; Zeman et al., 2009; Jeong et al., 2012; Wilhelm et al., 2013
Resistin	Indirect link to depression. The pathway is unknown	Krsek et al., 2004; Silha et al., 2004; Weber-Hamann et al., 2007; Lehto et al., 2010
Insulin	Linked to mood, depression, anxiety and negative emotion via insulin receptor signaling	Gustafson et al., 1999; Benedict et al., 2004; Koponen et al., 2008; Akbaraly et al., 2009; Almeida et al., 2009; Benoit et al., 2009; Kleinridders et al., 2009; Marks et al., 2009; Pulkki-Raback et al., 2009; Grillo et al., 2011; Chapman et al., 2013; Platt et al., 2013

Chocolate has a strong effect on mood, generally increasing pleasant feelings and reducing tension (Osman and Sobal, 2006; Parker et al., 2006b; Cartwright et al., 2007; Fletcher et al., 2007). Chocolate contains psychoactive chemicals such as andamines that stimulate the brain and result in good mood (Ottley, 2000). However, negative feelings are also associated with chocolate in some women on weight loss regimes who experience guilt after eating chocolate. The unique taste and feel from chocolate in the mouth leads to chocolate craving due to sensory factors associated with chocolate eating (Macht and Dettmer, 2006; Osman and Sobal, 2006; Parker et al., 2006b; Cartwright et al., 2007; Fletcher et al., 2007).

Caffeine, mostly consumed in the form of coffee and tea, not only has stimulant effects on enhancing alertness, vigilance, and reaction time but also increases anxiety in susceptible individuals (Acquas et al., 2002; Rossi et al., 2010). Caffeine blocks adenosine receptors in the brain and can relieve headaches, drowsiness, and fatigue. Short-term caffeine deprivation in regular users results in withdrawal symptoms (Rogers, 1995).

Omega-3 fatty acids, found in various foods can influence, mood, behavior, neuroticism, and impulse control (Van Strater and Bouvy, 2006; Conklin et al., 2007; Stahl et al., 2008). Omega-3 fatty acids play a role in major depressive disorder, bipolar disorder, schizophrenia, substance abuse, and attention deficit disorder (Young and Martin, 2003; Parker et al., 2006a; Van Strater and Bouvy, 2006; Stahl et al., 2008). Docosahexaenoic acid (DHA) and eicosapentaenoic acid (EPA), both members of the omega-3 fatty acid family, contribute to the fluidity of the cell membrane, and thereby play an important role in brain development and function (Pawels and Volterrani, 2008). Low blood levels of polyunsaturated omega-3 fatty acids are associated with depression, implying a role in mood disorders (Lombard, 2000; Sanchez-Villegas et al., 2007; Antypa et al., 2012; Moranis et al., 2012; Kang and Gleason, 2013; Grosso et al., 2014).

Micronutrients, such as thiamine (vitamin B1), iron, and folic acid, play a role in emotion. Thiamine containing foods influence mood states (Benton et al., 1995). Improved thiamine status increases well-being, sociability, and overall energy levels. Insufficient amounts of thiamine are associated with impaired mood and cognitive functioning (Benton et al., 1997; Benton and Donohoe, 1999).

Iron deficiency represents one of the most common nutritional problems worldwide. Iron deficiency anemia can result in depressed mood, and problems with attention and lethargy (Benton and Donohoe, 1999).

Folic acid plays an important role in the brain. Folic acid deficiency is associated with depressed mood (Coppen and Bolander-Gouaille, 2005; Young, 2007). Psychiatric patients often run the risk of developing folic acid deficiency due to loss of appetite from anticonvulsant drugs that inhibit folic acid absorption (Ottley, 2000). Collectively, these findings suggest foods influence mood.

Mood can influence food preference (Christensen and Brooks, 2006). Choice of eating palatable foods can either lead to comfort feeling or disgust. A good example of behavioral change that is observed after taking a meal is altered mood. A general effect of meal on behavior is observed from animals to humans where hunger leads to irritability and meal intake leads to arousal and alertness. Thus, a search for food is cultivated. Once satiety sets in, sedentary and calm behaviors most likely have positive rather than negative effect on mood (Macht and Simons, 2000; Macht et al., 2003; Macht and Dettmer, 2006; Macht, 2008). A potential internal information route on emotional behavior was first recognized in 2001 where nutrients from gut were relayed to the brain by the vagus nerve affecting emotions (Zagon, 2001). However, the relationship of emotions, physiological arousal, and mood in a given situation is significantly dependent upon on the subject's motivational state (Reid and Hammersley, 1999) and the individual's personality trait of neuroticism that interacts with mood and response to emotional stimuli (Dess and Edelheit, 1998).

The pathogenesis of both mood and metabolic disorders during obesity can be triggered by certain diets (Wallin and Rissanen, 1994; Sanchez-Villegas and Martinez-Gonzalez, 2013). Diets like Western diets that are rich in saturated fat and low in poly-unsaturated and mono-unsaturated fatty acids tend to increase the incidences of depression (Peet et al., 1998). On the other hand, diet like the Mediterranean diet appears to reduce depression (Sanchez-Villegas and Martinez-Gonzalez, 2013; Sanchez-Villegas et al., 2013). Furthermore, many reports show the increased incidence of depression on diets that lack omega-3 polyunsaturated fatty acids (PUFA) and that depression is reduced when intake of PUFA is increased in both humans (Lin and Su, 2007; Sanchez-Villegas et al., 2007; Oddy et al., 2011; Park et al., 2012a) and rodents (Moranis et al., 2012; Park et al., 2012b). Besides mood changes, high fat diets promote increased weight gain, visceral adipose tissue, larger waist circumference, and more cardiovascular disease mortality (Schulze et al., 2006; Molenaar et al., 2009; Romaguera et al., 2009, 2010; Mozaffarian et al., 2011; Estruch and Salas-Salvado, 2013; Nazare et al., 2013). The accumulation of adipose tissue in abdominal stores leads to several complications of obesity including insulin resistance leading to metabolic syndrome (Despres et al., 2006; Tchernof and Despres, 2013). These changes also lead to neurobiological impairments affecting mood disorders such as depression and anxiety (Weber-Hamann et al., 2002; Van Reedt Dortland et al., 2013a,b). It is believed that increased circulating plasma fatty acids such as palmitic acid enters the brain and impairs neurological function (Tsuboi et al., 2013). Palmitic acid impairs leptin and insulin receptor signaling in the hypothalamus and promotes weight gain (Benoit et al., 2009; Kleinridders et al., 2009). Under these circumstances, obesity is promoted, as well as a negative emotional state. In addition, leptin and insulin have been noted to influence mood (Gonder-Frederick La et al., 1989; Lu et al., 2006; Lu, 2007; Zeman et al., 2009; Ryan et al., 2012).

Furthermore, several studies have shown humans on high fat diet manifest mood disorders like depression that correlates positively with high serum palmitate (Tsuboi et al., 2013). Similarly, rats on high fat diet display increased anxiety-like behavior, altered body weight, plasma insulin, leptin, and glucose levels when compared to rats on iso-caloric olive oil high fat diet that show no changes in body weight, glycaemia, leptin, and insulin levels (Hryhorczuk et al., 2013). Thus, saturated fats stimulate HPA disturbances and/or inflammation, leading to anxiogenic-like behavior in animals and depression in humans. All together these findings suggest an association between certain foods and improved mood.

### Psychiatric and eating disorders

The Diagnostic and Statistical Manuals of Mental Disorders (DSM-5), which was developed by the American Psychiatric Association in 1994, reported disturbed eating behaviors in psychiatric disorders (American Psychiatric Association, 2013). In humans, melancholic depression is associated with hypercortisolism, anhedonia, hypophagia, and weight loss (Fisher et al., 1997; Krishnan and Nestler, 2008; Ulrich-Lai and Herman, 2009; Hammack et al., 2010; Carroll et al., 2012; Hryhorczuk et al., 2013; Patterson and Abizaid, 2013; Schellekens et al., 2013b). In contrast to atypical depression, the most common forms of depression are characterized by reduced hypothalamic pituitary adrenal axis (HPA) activity, increased appetite, carbohydrate craving, and weight gain (Juruena and Cleare, 2007). Those with abdominal obesity are associated with hyperactive HPA axis due to an elevated response to corticotrophin releasing hormone (CRH) stimulation and increased stimulated response to stress (Pasquali, 2012).

Altered serum cortisol level is associated with depression (Parker et al., 2003; Raison and Miller, 2003; Stetler and Miller, 2011). Altered cortisol, HPA axis, and food intake have been associated with depression (Ulrich-Lai and Herman, 2009; Dallman, 2010; Schellekens et al., 2012a). The neuronal pathways that regulate food intake, and circuitries that act *via* the HPA axis are implicated in a complex two-way relationship of three concepts between mood, food, and eating behavior (Figure 1) (Kyrou and Tsigos, 2009; Ulrich-Lai and Herman, 2009; Dallman, 2010; Schellekens et al., 2012b, 2013b). It is noted that there is an overlap in neural circuitry of food intake and stress that likely reinforces a link between stress and feeding behavior (Maniam and Morris, 2012). These overlapping circuitries of HPA axis modulating feeding behavior and stress converge on corticosterone hormone producing neurons in the paraventricular nucleus (PVN). Thus, elevated glucocorticoid and a dysfunctional HPA axis are common to both depression and obesity.

Glucocorticoids exert multiple effects on metabolic, endocrine, immune, and behavioral functions. Glucocorticoids regulate reward and emotional processes *via* their receptors in midbrain and limbic circuits (Arnett et al., 2011; Solomon et al., 2012; Hryhorczuk et al., 2013; Patterson and Abizaid, 2013; Wang et al., 2013). Glucocorticoids not only act peripherally to maintain energy homeostasis but also centrally to modulate HPA activity, emotional, and behavioral effects of stress (Fedoroff et al., 2003; Figueiredo et al., 2003). Under physiologic acute stress, the HPA axis is activated, and glucocorticoids are released. This leads to a major restoration of energy balance by increasing insulin, increasing motivation for palatable food (Piazza and Le Moal, 1997; Dallman et al., 2006; Dallman, 2010), and mobilizing stored energy toward central stores that leads to obesity (Mann and Thakore, 1999). Thus, obesity and mood disorder are linked *via* the HPA axis. In rodents, chronic corticosterone exposure leads to increased glucocorticoid receptor (GC) expression in fore-brain and basolateral amygdala that results in depressive-like, anxiety-like behaviors, and increased locomotors (Wei et al., 2004; Boyle et al., 2005, 2006). Therefore, these findings suggest that a deficit in glucocorticoid signaling in distinct brain regions may play a role in affective disorder.

### Obesity and mood

Obesity increases incidence of anxiety and mood disorders (Simon et al., 2006). Stress induced overeating and obesity is also associated with major depression in humans (Novick et al., 2005; Simon et al., 2006; Kloiber et al., 2007). Individuals under chronic stress tend to have more visceral fat due to excessive systemic cortisol levels (Brown et al., 2004; Adam and Epel, 2007; Kyrou and Tsigos, 2009). In all, there appears to be a good association between hypercortisolemic depression, abdominal fat accumulation (Weber-Hamann et al., 2002), decreased glucocorticoid-mediated negative feed back, and increased corticotropin releasing hormone (CRH) release from the paraventricular nucleus (PVN) (Holsboer, 2000). Furthermore, major depression in adolescence is linked to a higher risk for obesity in adulthood (Richardson et al., 2003). It is also noted that metabolic conditions are exacerbated in depression and vice versa (Mcelroy et al., 2004; Simon et al., 2006; De Wit et al., 2010; Luppino et al., 2010; Marijnissen et al., 2011). Like-wise, stress significantly impacts food intake in both humans and animals, thereby promoting metabolic disturbances (Block et al., 2009; Dallman, 2010; Maniam and Morris, 2012). Overeating can also be considered to be analogous to drugs of use because it reflects an addiction where individuals become physically and psychologically dependent on foods rich in fat and sugar (Avena et al., 2008, 2009; Barry et al., 2009; Parylak et al., 2011; Allen et al., 2012; Davis, 2013). Reports also show that with intake of palatable rewarding food, acute stress responses are reduced (Dallman et al., 2003; Lutter and Elmquist, 2009; Chuang et al., 2011; Kumar et al., 2013), thereby showing the potential of “comfort eating” in stress relief. All together these findings suggest that there is a reciprocal link in mood disorder and obesity.

### Rodent models of mood and eating disorders

Rodent studies have provided the best insight into dopamine-mediated food intake. Dopamine deficient mice die quickly due to decreased food intake (Hnasko et al., 2004). Dopamine when given in the striatum rescues deficient food intake by restarting feeding behavior. Further, when dopamine is given to the nucleus accumbens, a food preference for pleasant food vs. non-pleasant food is observed. Altered dopamine receptor expression is also associated with feeding behavior (Clifton et al., 1991; Zeng et al., 2004; Wang et al., 2009) (18). Post-transcriptional modification such as RNA editing could also play a role in altered reward circuitry mediating overeating behavior (18). It is noteworthy that altered serotonin 2C receptor (5HT_2C_R) editing has been associated with dopamine production, reward, mood, feeding, and recently obesity (Burns et al., 1997; Sodhi et al., 2001; Gurevich et al., 2002; Higgins and Fletcher, 2003; Iwamoto et al., 2005; Rosenzweig-Lipson et al., 2007; Berg et al., 2008; Olaghere Da Silva et al., 2010; Hayes and Greenshaw, 2011; Schellekens et al., 2012a). Intriguingly, both serotonergic and dopaminergic system are altered in transgenic mice with dysregulated RNA editing enzyme, ADAR2 (Singh et al., 2007, 2009, 2011) (18). These transgenic mice show significantly hyperactive brain regions implicated in reward and also behaviorally display goal oriented behavior toward food in a competitive rewarding environment (Akubuiro et al., 2013). Furthermore, altered dopamine receptor expression, food preference for high fat diet are also observed in ADAR2 transgenic mice (Akubuiro et al., 2013). Interestingly, co-morbidities of depression and anxiety behaviors and altered 5HT_2C_R editing are observed in ADAR2 transgenic mice (Singh et al., 2007, 2009, 2011). Collectively, these results suggest that co-morbidities of affective disorder, overeating, and obesity could be linked *via* the modified 5HT_2C_R in ADAR2 transgenic mice. However, more studies are required to provide a better understanding of the post-transcription modification of the 5HT_2C_R linking to mood, food, and obesity in ADAR2 transgenic mice.

Dysfunctional serotonergic signaling has been associated with mood and obesity (Wurtman and Wurtman, 1989; Benton and Donohoe, 1999; Sodhi et al., 2001; Iwamoto and Kato, 2003; Schmauss, 2003; Kawahara et al., 2008; Morabito et al., 2010; Singh et al., 2011; Schellekens et al., 2012a; Silberberg et al., 2012; Shinozaki et al., 2013). In another rodent model of depression brain derived neurotropic factor (BDNF) was shown to have an antidepressant-like effect (Siuciak et al., 1997). BDNF has been shown to be a neurotropic factor on serotonergic neurons in BDNF heterozygous mice where dysfunctional serotonergic signaling is associated with aggression, hyperphagia, and weight gain is rescued (Lyons et al., 1999). Further exogenous BDNF application enhances serotonin signaling and modifies several behaviors regulated by serotonin feeding, body weight homeostasis, and analgesia (Siuciak et al., 1994; Pelleymounter et al., 1995). Thus, these studies suggest that dysfunctional serotonergic and dopaminergic systems play a critical role in mood, food intake, and obesity.

### Psychobiological relationship of brain reward linking hunger, addiction, overeating, and obesity

Continuous overeating can be viewed as an addictive behavior that involve reward circuitry (Davis, 2013). Reward circuitry involved in addiction spans two key brain regions, (1) the prefrontal region and the amygdala and, (2) the limbic system integrating amygdala with hypothalamus and septal nuclei (Elliott et al., 2000; Schultz, 2000, 2002; Tzschentke, 2001; Baxter and Murray, 2002; Rolls et al., 2002; Koob and Volkow, 2010). The neural mechanism of disrupted dopamine signaling pathways being central to overeating and drugs of use and the overwhelming hallmarks of urge to seek and consume, thereby presents an addiction behavior. Another common phenomenon of compulsive intake of drugs and overconsumption of food intake seen in obesity is the loss of control due to impairments in circuits involved in decision making, self control, interoception, and regulation of mood and stress (Volkow et al., 2010).

Two hormones: ghrelin and leptin interact with the hypothalamus to regulate food intake, energy homeostasis, promote satiety, and hunger. Interestingly, both hormones have been implicated in craving behavior, eating disorder, and mood and have also been associated with the reward pathway (Kiefer et al., 2001; Opland et al., 2010; Dickson et al., 2011). Thereby, suggesting that both ghrelin and leptin are linked to mood and food intake.

There are several neurotransmitter systems involved in feeding such as serotonin, dopamine, opioids, and GABA, of which serotonin and dopamine have been the most closely linked to feeding behavior. Dopamine mediates reward specifically the “wanting” or approach behaviors toward a biologically relevant goals more so than “liking” or enjoyment aspect (Berridge, 1996; Davis et al., 2009). Opioids have been implicated more so in the “liking” or the hedonic aspect of reward processing and both neurotransmitter pathways work together in the perception of reward (Davis et al., 2009). The “wanting” behavior toward a biological relevant goal that is mediated by dopamine is probably due to how dopamine neurons receive signals and the way they are organized in the brain. Dopamine neurons are found in the midbrain region of the ventral tegmental area (VTA) and substantia nigra pars compacta projecting to striatal limbic and cortical regions. Dopamine neurons receive information from; hypothalamus and brain stem regions involved in autonomic responses, hippocampus involved in memory, amygdala involved in emotional reactivity, thalamus involved in arousal and prefrontal cortex and cingulate involved in emotional reactivity *via* neuropeptides and neurotransmitters. Neurochemistry and neuroanatomical reward circuitry involved in addiction to alcohol and drugs translate to an addiction model of overeating and obesity. Certain studies show that hunger can influence memory for food-related stimuli where the orbitofrontal cortex is specifically involved in food-related stimuli in hunger state (Morris and Dolan, 2001). In rodent studies, dopamine has been shown to play a role in feeding by determining a meal size to meal duration, and obesity (Clifton et al., 1991; Schwartz, 2000). Dopamine in the nucleus accumbens has been associated with reinforcement aspects of food and while in the hypothalamus, dopamine plays a role in initiation and duration of feeding (Wang et al., 2004b). Leptin and insulin also help to regulate dopamine production (Leinninger et al., 2009). Dopamine regulates food consumption involving the mesolimbic pathway and the hypothalamus (Volkow et al., 2011). Since dopamine levels in addiction change in these brain regions, it is conceivable that a similar mechanism of reinforcement of food may also be involved in food addiction (Wang et al., 2004b).

### Food reward, addiction, and obesity

Food is a natural reward and has both homeostatic and hedonic characteristics (Rada et al., 2010; Volkow et al., 2011). Depending on the specific type of highly palatable food, it has the potential to engage similar brain reward pathways as drugs of abuse (Weatherford et al., 1990; Pitchers et al., 2010; Olsen, 2011). It may also arise from casual eating to compulsive eating that eventually leads to addiction (Davis, 2013). This may be from food-related brain changes that is associated with psychological changes like that seen in drug addiction (Robinson and Berridge, 2003). Both rewarding and hedonic effects of food result in positive emotional reactions that play a major role in overeating and obesity (Fulton, 2010; Avena et al., 2013; Bongers et al., 2013; Sinha and Jastreboff, 2013; Yau and Potenza, 2013). Theoretical models support food addiction because highly palatable food activates reward pathways that lead to human and animal obesity (Finlayson et al., 2007; Berner et al., 2008; Heyne et al., 2009; Davis et al., 2011; Sampey et al., 2011; Akubuiro et al., 2013; Davis, 2013).

The American Psychological Association in the DSM-5 manual included behavioral addiction and addictions to natural rewards as a new category of “addiction and related behavior” (Volkow and O'brien, 2007). Human and rodent studies suggest that dysregulated brain reward pathways may contribute to increased intake of palatable food leading to obesity (see review by Berthoud et al., 2011). Despite the divergence in eating behavior, there is an overall increase in tasty, energy- rich foods that is independent of stress-induced hyperphagia or hypophagia (Gibson, 2006; Dallman, 2010). One hallmark of food addiction is the food craving where intense desired food consumption only compensates the craving, whereas in hunger various types of food alleviates the hunger (Martin et al., 2011). Advantages of functional magnetic resonance imaging (fMRI) and positron emission topography (PET) paradigms have been used to provide insights of neural correlates in food addiction and obesity (Wang et al., 2004a; Teegarden and Bale, 2007; Volkow et al., 2012). Interestingly, following various types of food presentation to normal healthy patients, activated brain regions of anterior cingulate cortex, orbitofrontal cortex, and insula are observed (Wang et al., 2004a; Teegarden and Bale, 2007). In contrast to obese overeating patients, neurobiological changes in the reward pathways are similar to those observed in drug addicts (Volkow et al., 2012). However, available data in humans on food addiction suggests that there is heterogeneity in the clinical definitions of food addiction, obesity, and binge eating disorder. Nonetheless through neurobiological data obtained from both human and animal studies, food cravings, overeating, and tolerance support an addiction-like model, see reviews (Albayrak et al., 2012; Volkow et al., 2012; Davis, 2013; Hone-Blanchet and Fecteau, 2014).

### Society and food addiction

Globally about 1 billion adults are overweight of which 475 million are obese (Organization, 2013). Obesity is a complex multifactorial disease. In the United States, increased incidence of adult obesity is on the rise. In the Westernized society, the major cause of obesity is due to reduced physical activity leading to sedentary life style and surplus of food, sodas, variety of fast food, and hyperpalatable foods, all that activate dopamine rewarding centers leading to over consumption of food (Fortuna, 2012; Granados et al., 2012; Ziauddeen et al., 2012). Hyper-palatable foods and their increased availability promote addictive and compulsive eating leading to weight gain. Addictive properties of certain types of food and addiction-like behaviors are observed in both humans and animal models. Animal studies have shown an overview of addiction-like eating behaviors when presented with foods high in sugar and fat (Avena et al., 2008, 2012). In animals, several studies of sugar-binging models support an addiction-like phenotype of tolerance, cross sensitization, withdrawal, and neurochemical changes, but does not induce obesity (Avena, 2007; Avena et al., 2008, 2009). On the other hand, several imaging studies from obese population shows that greater BMI and overeating are associated with neurobiological pathways similar to those observed in drug addicts (Stice and Dagher, 2010; Stice et al., 2010; Volkow et al., 2012, 2013). In humans, feeding behaviors are more complex but pattern of food addiction appears to parallel substance dependence (Gearhardt et al., 2011; Dileone et al., 2012). Some argue that food addiction should be included in the DSM manual (Volkow and O'brien, 2007; Taylor et al., 2010) even though food addiction is not a categorized diagnosis within DSM-5. However, recently Yale Food Addiction Scale (YFAS) has been used as a tool for diagnosis of food addiction in patients with eating disorders (Gearhardt et al., 2009; Clark and Saules, 2013). In one study, using body mass index, body fat percentage by dual-energy X-ray absorptiometry, macronutrient intake, and the YFAS scale has been used as a diagnostic tool to assess food addiction in general Newfoundland population (Pedram et al., 2013). They found that the prevalence of food addiction was significantly associated with obesity in general population. Thus, suggesting that food addiction contributes to severity of obesity in the general population and that food addiction could be a separate etiology of obesity.

In summary, findings of central mediated food intake suggest a complex two-way link between food intake and mood, emotion, reward, food, food choice, and neurotransmitters (Figure 1). Food addiction remains as an incomplete described phenomenon due to limited data. Overabundance of food seems to aid in food addiction specifically foods rich in fat and sugar. Although FMRI and PET imaging have been useful in providing some insights into neural correlates in food addiction and obesity, but specific food addiction phenotype in the development of obesity needs to be differentiated. Furthermore, molecular pathways or signatures that link food intake in emotion, mood, food, reward, and obesity are areas that need further investigation. These types of studies in the future will provide further insight into genetic, psychological, neuropsychiatric, and environmental risk factors associated with overeating, food addiction, and obesity.

## Peripheral system in regulation of mood, food, and obesity

The gut-brain axis mediates the communication between brain and gut when it comes to appetite, satiety, and energy homeostasis (Cummings and Overduin, 2007; Ahima and Antwi, 2008; Blevins and Baskin, 2010; Gibson et al., 2010; Suzuki et al., 2010, 2012). Furthermore, peripheral hormones have also been reported to regulate mood, food intake, and obesity (Tschop et al., 2000; Nakazato et al., 2001; Olszewski et al., 2008; Blevins and Baskin, 2010; Suzuki et al., 2010; Andrews, 2011b; Dickson et al., 2011; Egecioglu et al., 2011; Skibicka and Dickson, 2011; Overduin et al., 2012; Perello and Zigman, 2012; Karra et al., 2013). Gastrointestinal signals such as cholecystokinin (CCK), bombesin, glucagon eneterostatin, insulin, resistin, somatedin, cyclohistiyl-proline, leptin, amylin, and apolipoprotein A-IV are all known to reduce food intake. The exception is ghrelin, which increases food intake. Several peripheral factors that engage the CNS in a bi-directional manner and influence mood and food intake are summarized in Table 1 and discussed below.

### Ghrelin

A gut orexigenic hormone ghrelin is synthesized in the stomach and acts centrally to mediate increased food intake *via* central pathways (Kojima et al., 1999, 2004; Tschop et al., 2000; Nakazato et al., 2001; Andrews, 2011a; Diz-Chaves, 2011). The hypothalamus in the brain directly senses peripheral ghrelin and modifies the energy status (Schaeffer et al., 2013). Studies support that ghrelin reaches the brain *via* the vagus afferents to the nucleus solitary tract (NST), which further projects to the arcuate nucleus of the hypothalamus (Asakawa et al., 2001; Date et al., 2002; Williams and Mobarhan, 2003). Ghrelin activates downstream signaling *via* the hormone secretagogue receptor (GSH-R1a) where it is ubiquitously expressed in multiple brain regions and in peripheral tissues. Due to multiple sites of GSH-R1a expression, it is not surprising that ghrelin performs many other biological activities of growth hormone secretion, glucose and lipid metabolism, and gastrointestinal motility. However, other properties of GHS-R1a allowing dimerization with multiple G-protein coupled receptors suggest the likelihood of cross talk between many other neuropeptide systems of serotonin and dopamine (Schellekens et al., 2013a,b). Thus, ghrelin has the potential to engage multiple neuropeptide systems in mood, food, and obesity.

The ghrelinergic system also mediates the non-homeostatic hedonic rewarding and motivational aspects of food intake *via* mesolimbic dopaminergic circuitry (Dickson et al., 2011; Egecioglu et al., 2011; Skibicka et al., 2011; Perello and Zigman, 2012). Studies support ghrelin's involvement in stress mediated food reward behavior (Perello et al., 2010; Kumar et al., 2013; Chuang et al., 2011; Diz-Chaves, 2011). Numerous studies provide a link between ghrelin and affective disorders, such as depression and anxiety (Schanze et al., 2008; Barim et al., 2009; Kluge et al., 2009). Ghrelin also alleviates depression (Kluge et al., 2011). All together these studies suggest that the ghrelinergic system is an attractive system to target stress associated metabolic and mood associated eating disorders in obesity.

### Serotonin

Serotonin has numerous functions besides regulating mood that includes regulation of sleep, appetite, and impulse control (Steiger, 2004; Daubert and Condron, 2010; Nordquist and Oreland, 2010; Mosienko et al., 2012). Serotonin levels from the gut and alimentary canal constitutes about 80–90% of the human body's total serotonin and not in the brain. This is surprising, as serotonin dictates most of our mood and happiness (Wurtman and Wurtman, 1989; Benton and Donohoe, 1999). Central serotonin pathways participate in the regulation of mood and modulate meal patterns in terms of quality and quantity. Neurotransmitter release of serotonin from serotonergic neurons in the brain is governed by food intake (Shabbir et al., 2013). The essential amino acid tryptophan that comes from food is the precursor for serotonin synthesis (Prasad, 1998). Ingestion of carbohydrates increases the plasma ratio of tryptophan to other large neutral amino acids leading to increased serotonin synthesis in the brain and alleviating depression. Such is the case for carbohydrate craving during depression that often leads to obesity and vice versa (Pepino et al., 2009; Shabbir et al., 2013). This is observed during stress, winter depression, or in people trying to give up smoking. Nicotine increases brain serotonin secretion and its withdrawal leads to depression (Wallin and Rissanen, 1994; Wurtman and Wurtman, 1996). Brain serotonin plays a role in the pathophysiology of depression, as treatments with serotonin potentiating drugs alleviates depression in seasonal affective disorder (Wurtman, 1993). Based on these findings it has been suggested that the excessive carbohydrate intake by patients with premenstrual syndrome (PMS) and seasonal affective disorder (SAD) relieves the depressive symptoms *via* an increased central serotonergic activity (Cizza et al., 2005; Miller, 2005). A diet rich in carbohydrates can relieve depression and elevate mood (Wurtman and Wurtman, 1989; Benton and Donohoe, 1999). Furthermore, research has shown that dieters tend to become depressed as the serotonin levels are reduced due to decreased carbohydrate intake (Huether et al., 1997). Thus, these studies imply that certain foods are strong mood regulators.

### Leptin

Low leptin levels have been found to be associated with human depression and depression-like behaviors in rodents (Kraus et al., 2001; Lu et al., 2006; Guo et al., 2012; Lawson et al., 2012). Antidepressant-like effect of leptin in leptin insufficiency or leptin resistance suggests the hormone contributes to altered mood (Lu, 2007). Increased visceral fat and dyslipidemia are associated with several endocrine and metabolic changes that link to CNS control of emotional states and mood (Hryhorczuk et al., 2013). As an endocrine gland, adipose tissue secretes numerous peptide hormones that target the brain and peripheral tissues to regulate metabolism and behavior. Leptin circulates in proportion to fat mass (Maffei et al., 1995). Leptin impacts several physiological processes such as appetite, energy expenditure, and neuroendocrine function. The hormone has also been linked to human depression and has been shown in rodents to have antidepressant and anxiolytic effects (Asakawa et al., 2003; Liu et al., 2010; Yamada et al., 2011; Lawson et al., 2012). Nevertheless, there are conflicting findings of leptin levels and depression, which are discussed below.

Major depressive disorder (MDD) has been shown to be associated with lower plasma leptin levels when compared to healthy controls (Kraus et al., 2001; Atmaca et al., 2002, 2008; Westling et al., 2004; Jow et al., 2006). On the other hand, there are reports showing increased plasma leptin levels in depression (Kraus et al., 2002; Esel et al., 2005; Schilling et al., 2013), gender specific increased leptin levels in women with depressive disorder (Rubin et al., 2002; Esel et al., 2005; Zeman et al., 2009), as well as no changes of leptin by antidepressant treatment (Esel et al., 2005). In depressed individuals suffering from loss of appetite, plasma leptin levels do not differ from those of healthy controls (Deuschle et al., 1996). In another study, it was found that higher serum leptin was associated with atypical depressive patients with increased appetite (Gecici et al., 2005). In older men, a combination of elevated visceral fat and high leptin levels was associated with depression (Milaneschi et al., 2012), and high leptin correlated positively with depressive symptoms in patients with type 2 diabetes (Labad et al., 2012). Thus, these reports suggest more studies are required to draw a better conclusion regarding the role of leptin in human depression.

Interestingly, rodent studies have provided the most conclusive findings. Leptin modulates the HPA axis and mice that lack leptin (obese *ob/ob* mice or its leptin receptor (obese *db/db* mice) show increased depression-like behavior (Collin et al., 2000; Asakawa et al., 2003; Lu et al., 2006; Finger et al., 2010; Liu et al., 2010; Sharma et al., 2010; Yamada et al., 2011; Guo et al., 2012, 2013). Furthermore, leptin deficient *ob/ob* mice have elevated corticosterone that can be reduced by leptin replacement (Garthwaite et al., 1980; Arvaniti et al., 2001). In contrast, chronic unpredictable mild stress in rats activates the HPA axis and leads to depressive-like behaviors that correlate with decreased serum leptin levels (Ge et al., 2013). Leptin receptors (LepRb) in midbrain and forebrain loci that affect emotional processes are targeted by leptin. Genetic deletion of LepRb in the hippocampus results in a depression-like phenotype, which is reduced by leptin administration to the hippocampus thereby showing an antidepressant effects (Asakawa et al., 2003; Lu et al., 2006; Finger et al., 2010; Liu et al., 2010; Guo et al., 2013). Loss of LepRb specifically in glutamatergic neurons of the forebrain elicits depressive-like behavior without affecting anxiety (Guo et al., 2012). Stress-induced dopamine release is also associated with high leptin (Burghardt et al., 2012). Leptin activates dopamine neurons in the VTA of the midbrain reducing dopamine neuronal firing and increases dopamine availability (Fulton et al., 2006; Hommel et al., 2006). Selective deletion of LepRb from midbrain dopamine neurons results in increased anxiety-like behavior, but not depressive-like behavior (Liu et al., 2011). LepRb signaling in limbic and prefrontal nuclei mediates the antidepressant action of leptin. In contrast, leptin in dopamine neurons of the ventral midbrain and in central nucleus of the amygdala leptin signaling exerts the anxiolytic actions of leptin. Thus, leptin signaling in different brain regions exerts different physiological behaviors.

In conditions of central obesity that favors insulin resistance and type 2 diabetes, leptin sensitivity is diminished. Leptin resistance is associated with high plasma leptin levels and defective LepRb signaling. These states are characteristic of obesity and increase the risk for mood disorders (Myers et al., 2012). Mice made obese by a high fat diet intake show reduced sensitivity to effects of leptin and antidepressant actions of leptin when compared to low-fat diet treated controls (Yamada et al., 2011). Further, leptin insensitivity exacerbates HPA dysregulation in obesity (Komorowski et al., 2000; Collura et al., 2009) and thereby enhances the mass of dysfunctional central adipose stores in a cortisol-dependent manner. Leptin resistance has been reported to be associated with the mid brain VTA where mesolimbic DA neurons reside (Matheny et al., 2011). Leptin resistance appears to affect multiple neural and endocrine pathways including hippocampal, mesolimbic dopamine pathways, and HPA activity ultimately affecting emotions and mood. Thus, these studies provide evidence of leptin related mechanisms underlying depression in obesity.

### Adiponectin

Low levels of another adipose-derived hormone, adiponectin, has been implicated in energy homeostasis, metabolic disturbances, insulin resistance (Kennedy et al., 2006; Hanley et al., 2007; Turer and Scherer, 2012; Hryhorczuk et al., 2013) and recently, depression in humans (Arita et al., 1999; Cnop et al., 2003; Ryo et al., 2004; Leo et al., 2006; Narita et al., 2006; Hanley et al., 2007; Weber-Hamann et al., 2007; Yilmaz, 2008) and rodents (Maeda et al., 2001; Milan et al., 2002; Delporte et al., 2004; Ye et al., 2007). Changes in adiponectin levels are secondary to metabolic disturbances in obesity (Morrison et al., 2011; Doumatey et al., 2012). There are conflicting reports of either positive or negative associations of adiponectins levels with mood disorder (Yilmaz, 2008; Zeman et al., 2009; Jeong et al., 2012; Wilhelm et al., 2013), or no changes in patients with major depressive disorder or with antidepressants (Lehto et al., 2010; Jeong et al., 2012). Mice exposed to chronic social defeat recapitulate the low levels of adiponectin, stress-induced depressive-like behaviors, and impaired HPA axis (Liu et al., 2012). Interestingly central administration of adiponectin has antidepressant effects (Liu et al., 2012). Thus, a link between plasma adiponectin levels and depression is observed in mice. In contrast, humans show more ambiguous results depending on the type of depressive disorder, sex, and treatment.

### Resistin

Adipocyte-derived resistin is linked to insulin resistance in rodent models of depression-like behavior while in humans, the role of resistin is less defined (Schwartz and Lazar, 2011; Hryhorczuk et al., 2013). In genetic and diet induced obese mice circulating resistin levels are elevated (Steppan et al., 2001). In contrast, resistin is down regulated in human obesity (Way et al., 2001; Degawa-Yamauchi et al., 2003; Owecki et al., 2011; Sadashiv et al., 2012). However, there is one study that shows a positive correlation between resistin levels and atypical depression (Lehto et al., 2010). In human depression, however, resistin levels positively correlate with salivary cortisol (Krsek et al., 2004; Silha et al., 2004; Weber-Hamann et al., 2007). Conversely, resistin levels are lower in patients receiving antidepressant treatment who have remitted from depression (Weber-Hamann et al., 2007). Thus, these studies imply that resistin plays a role in affecting mood.

### Insulin

From a recent systematic review and meta-analysis there appears to be a significant cross-sectional association between depression and insulin resistance (Kan et al., 2013) and there is a bi-directional association between diabetes and depressed mood. Depression is associated with pre-diabetes insulin resistance (Anderson et al., 2001; Kan et al., 2013) and obesity (Hamer et al., 2012). However, there exists a weak association of insulin resistance and depression (Adriaanse et al., 2006; Platt et al., 2013; Shen and Bergquist-Beringer, 2013). High fat diet intake impairs the hypothalamic insulin receptor signaling (De Souza et al., 2005; Kim and Feldman, 2012) and reduced hypothalamic insulin signaling promotes weight gain and negative emotional states (Gustafson et al., 1999; Koponen et al., 2008; Akbaraly et al., 2009; Almeida et al., 2009; Benoit et al., 2009; Kleinridders et al., 2009; Pulkki-Raback et al., 2009; Platt et al., 2013). Intranasal insulin ameliorates self-reported mood, reduce cortisol levels, and visceral obesity (Benedict et al., 2004; Chapman et al., 2013). Further treating patients with major depressive disorder and abdominal obesity, the insulin-sensitizing drug pioglitazone shows reduced sign of depression, anxiety, and reduced insulin resistance (Kemp et al., 2012).

In rodents, reduced insulin receptor signaling impacts mood when placed on a long-term 30%kcal fat diet that shows anxiolytic effects (Marks et al., 2009). Similarly, rosiglitazone administered to normal chow-fed mice and rats show an antidepressant action in behavioral despair tests (Eissa Ahmed et al., 2009; Ryan et al., 2012). Antisense RNA targeting the insulin receptor in rats results in increased depression-like behavior and anxiety-like behavior (Grillo et al., 2011). By and large, these results suggest that insulin signaling is involved in mood. However, further studies are required to determine whether intranasal insulin has antidepressant effects in depressed individuals and, if so, whether this action is maintained in obesity.

To summarize, food intake is regulated by the peripheral and central system that are engaged in a bi-directional manner. Peripheral signals mostly modulate satiety and indicate adiposity signal to the brain. Ghrelin is the only peripheral hormone that induces hunger but interestingly it is also involved in mood and hedonic aspects of food intake. There are several brain regions involved in food intake that overlaps brain areas involved in drugs of abuse and reward. Overlapping brain regions of reward, mood, and food intake suggests that molecular changes in these regions may provide further insights in to distinct and overlapping pathways that could aid in understanding clinical treatments of comorbidity of mood disorder, overeating, and obesity.

## Epigenetics, mood, and eating disorder

Interaction of genes and environment has been associated with mood disorders, see review Archer et al. (2013), and eating disorders, see review Pjetri et al. (2012). Exposure to highly palatable foods rich in fat and carbohydrate induces craving. In an obesogenic environment, repetitive exposures to highly palatable food options increase the likelihood of food addiction, overeating, and obesity. There appears to be a complex interaction between genetics and environmental factors such as nutrition with neuropsychiatric, neurodevelopmental, and neurodegenerative disorders. Individual variability in numerous protein coding and non-coding regions in the genome could be related to eating disorders and affective disorders. Epigenetics mechanisms of DNA methylation, RNA editing, post-translational modification of histones, and non-coding RNAs regulate gene regulation without changing DNA sequence in response to changes in internal and external environmental variables. Epigenetics in the context of eating disorders is interesting as it has the potential to answer numerous questions including potential risk factors such as maternal nutrition and stress that alter the risk of eating disorders in the offspring. Unknown questions like how epigenetic modification responds to acute changes like malnutrition or exposure to highly palatable food needs to be answered. Furthermore, epigenetics in learning and memory could also play a critical role in development and maintenance of eating disorders. RNA editing of the 5HT_2C_R has been implicated in affective disorder, stress, maternal separation, Prader Willi Syndrome, hyperphagia, and obesity (Iwamoto and Kato, 2003; Englander et al., 2005; Iwamoto et al., 2005; Bhansali et al., 2007; Kawahara et al., 2008; Morabito et al., 2010; Singh et al., 2011; Schellekens et al., 2012a, 2013b,c). RNA editing of the 5HT_2C_R alters many facets of serotonin signaling *via* 24 different receptor isoforms. These edited 5HT_2C_R isoforms are in a distinct ratio in different brain regions suggests an important role in linking mood, food intake and obesity *via* the 5HT_2C_R. Therefore, future research in defining the role of different isoforms in different brain regions is much needed to understand the regulation of RNA editing and mood disorder by the serotonergic system.

## Conclusion

More than a third of adults and 17% of children and teenagers in the United States are obese (Ogden et al., 2014). Obesity is the second-leading cause of preventable death in the U.S. contributing to 300,000 deaths each year. In addition, the health care burden in obesity-related diseases in the U.S. could reach at staggering $861–957 billion by 2030 (Go et al., 2013a,b, 2014). This article points to biological factors engaging both central and peripheral system in a bi-directional manner linking food intake, mood, and obesity. Food intake is complex due to influence of several factors. The influence of food choice includes biological determinants of hunger, appetite, and taste. Besides these, other factors of cost, income, and availability also influence food choice. Other determinants of social and psychological factors of mood, stress, and emotion also play a critical role in food choice. Many people find it hard to stop eating a particular food even though they are not hungry. Such behaviors activate the brain reward center and alter the brain structure. Willpower has been speculated in the past to control overeating. Through neurobiological data, presence of food cravings, over eating, and tolerance support an addiction-like model by numerous signals that are involved in engaging both the central and peripheral nervous system in a bi-directional manner to regulate food intake. Genes, environment, various emotions also influence food intake, and mood states that trigger eating of palatable foods for comfort in negative emotional states. This repetitive eating of comfort foods, rich in carbohydrate, high-fats and sugar, leads to obesity. Obesity in turn regulates mood due to metabolic disturbances. Metabolic disturbances further alter brain-signaling systems leading to a bi-directional vicious cycle of mood, food, and obesity (Figure 1). Furthermore a complex regulation of mood and eating disorders are implied from emerging studies of epigenetics in mood and eating disorders.

### Future directions

It is recognized that animal and human findings do not entirely overlap, but animal studies have provided the most compelling neurobiological findings of addictive nature of food, overeating, food addiction, and obesity. In the future, using molecular studies toward an effort to understand the environment of plentiful food leading to obesity rather than food restriction in animal models will provide a valuable insight into the molecular mechanism of overeating and food addiction. Further, using animal models and molecular studies in the area of withdrawal induction model in highly palatable diet are needed. Understanding how brain regions are altered with various nutrients, in depression, anxiety state may elucidate a common overlapping brain region in co-morbidities of affective and eating disorders. Epigenetic progress in relation to eating disorder has been slow. Molecular pathways of regulated non-coding RNAs in gene regulation involved in affective disorders and overeating may provide novel pathways involved in the pathogenesis. Epigenetic regulation of primary brain signaling and factors governing their metabolism needs further investigation where animal studies are likely to guide psychiatric analysis of epigenetic modification. Furthermore, next generation sequencing can be useful in finding novel long and small non-coding RNAs, alternative spliced RNAs, expression levels of coding RNAs, and RNA editing changes in the clinical treatment responders vs. non-treatment responders. It is anticipated these future studies will aid in the development of more targeted and effective therapies for preventing and treating comorbidities of mood disorder and obesity.

### Conflict of interest statement

The authors declare that the research was conducted in the absence of any commercial or financial relationships that could be construed as a potential conflict of interest.

## References

[B1] Acquas E. Tanda G. Di Chiara G. (2002). Differential effects of caffeine on dopamine and acetylcholine transmission in brain areas of drug-naive and caffeine-pretreated rats. Neuropsychopharmacology 27, 182–193 10.1016/S0893-133X(02)00290-7 12093592

[B2] Adam T. C. Epel E. S. (2007). Stress, eating and the reward system. Physiol. Behav. 91, 449–458 10.1016/j.physbeh.2007.04.011 17543357

[B3] Adriaanse M. C. Dekker J. M. Nijpels G. Heine R. J. Snoek F. J. Pouwer F. (2006). Associations between depressive symptoms and insulin resistance: the Hoorn Study. Diabetologia 49, 2874–2877 10.1007/s00125-006-0500-4 17066302

[B4] Ahima R. S. Antwi D. A. (2008). Brain regulation of appetite and satiety. Endocrinol. Metab. Clin. North Am. 37, 811–823 10.1016/j.ecl.2008.08.005 19026933 PMC2710609

[B5] Akbaraly T. N. Kivimaki M. Brunner E. J. Chandola T. Marmot M. G. Singh-Manoux A. (2009). Association between metabolic syndrome and depressive symptoms in middle-aged adults: results from the Whitehall II study. Diabetes Care 32, 499–504 10.2337/dc08-1358 19106378 PMC2646036

[B6] Akubuiro A. Bridget Zimmerman M. Boles Ponto L. L. Walsh S. A. Sunderland J. Mccormick L. (2013). Hyperactive hypothalamus, motivated and non-distractible chronic overeating in ADAR2 transgenic mice. Genes Brain Behav. 12, 311–322 10.1111/gbb.12020 23323881 PMC4589229

[B7] Albayrak O. Wolfle S. M. Hebebrand J. (2012). Does food addiction exist? A phenomenological discussion based on the psychiatric classification of substance-related disorders and addiction. Obes. Facts 5, 165–179 10.1159/000338310 22647300

[B8] Allen P. J. Batra P. Geiger B. M. Wommack T. Gilhooly C. Pothos E. N. (2012). Rationale and consequences of reclassifying obesity as an addictive disorder: neurobiology, food environment and social policy perspectives. Physiol. Behav. 107, 126–137 10.1016/j.physbeh.2012.05.005 22583861 PMC3409327

[B9] Allison D. B. Newcomer J. W. Dunn A. L. Blumenthal J. A. Fabricatore A. N. Daumit G. L. (2009). Obesity among those with mental disorders: a National Institute of Mental Health meeting report. Am. J. Prev. Med. 36, 341–350 10.1016/j.amepre.2008.11.020 19285199

[B10] Almeida O. P. Calver J. Jamrozik K. Hankey G. J. Flicker L. (2009). Obesity and metabolic syndrome increase the risk of incident depression in older men: the health in men study. Am. J. Geriatr. Psychiatry 17, 889–898 10.1097/JGP.0b013e3181b047e3 19910877

[B21] American Psychiatric Association. (2013). Diagnostic and Statistical Manual of Mental Disorders. 5th Edn. Arlington, VA: American Psychiatric Publishing

[B11] Anderson R. J. Freedland K. E. Clouse R. E. Lustman P. J. (2001). The prevalence of comorbid depression in adults with diabetes: a meta-analysis. Diabetes Care 24, 1069–1078 10.2337/diacare.24.6.1069 11375373

[B12] Andrews Z. B. (2011a). Central mechanisms involved in the orexigenic actions of ghrelin. Peptides 32, 2248–2255 10.1016/j.peptides.2011.05.014 21619904

[B13] Andrews Z. B. (2011b). The extra-hypothalamic actions of ghrelin on neuronal function. Trends Neurosci. 34, 31–40 10.1016/j.tins.2010.10.001 21035199

[B14] Antypa N. Smelt A. H. Strengholt A. Van Der Does A. J. (2012). Effects of omega-3 fatty acid supplementation on mood and emotional information processing in recovered depressed individuals. J. Psychopharmacol. 26, 738–743 10.1177/0269881111424928 22004690

[B15] Archer T. Oscar-Berman M. Blum K. Gold M. (2013). Epigenetic modulation of mood disorders. J. Genet. Syndr. Gene Ther. 4:1000120 10.4172/2157-7412.1000120 23565345 PMC3615441

[B16] Arita Y. Kihara S. Ouchi N. Takahashi M. Maeda K. Miyagawa J. (1999). Paradoxical decrease of an adipose-specific protein, adiponectin, in obesity. Biochem. Biophys. Res. Commun. 257, 79–83 10.1006/bbrc.1999.0255 10092513

[B17] Arnett M. G. Kolber B. J. Boyle M. P. Muglia L. J. (2011). Behavioral insights from mouse models of forebrain–and amygdala-specific glucocorticoid receptor genetic disruption. Mol. Cell. Endocrinol. 336, 2–5 10.1016/j.mce.2010.11.011 21094675 PMC3172614

[B18] Arvaniti K. Huang Q. Richard D. (2001). Effects of leptin and corticosterone on the expression of corticotropin-releasing hormone, agouti-related protein, and proopiomelanocortin in the brain of ob/ob mouse. Neuroendocrinology 73, 227–236 10.1159/000054639 11340336

[B19] Asakawa A. Inui A. Inui T. Katsuura G. Fujino M. A. Kasuga M. (2003). Leptin treatment ameliorates anxiety in ob/ob obese mice. J. Diabetes Complicat. 17, 105–107 10.1016/S1056-8727(02)00185-X 12614977

[B20] Asakawa A. Inui A. Kaga T. Yuzuriha H. Nagata T. Fujimiya M. (2001). A role of ghrelin in neuroendocrine and behavioral responses to stress in mice. Neuroendocrinology 74, 143–147 10.1159/000054680 11528215

[B22] Atmaca M. Kuloglu M. Tezcan E. Ustundag B. (2008). Serum leptin and cholesterol values in violent and non-violent suicide attempters. Psychiatry Res. 158, 87–91 10.1016/j.psychres.2003.05.002 18155776

[B23] Atmaca M. Kuloglu M. Tezcan E. Ustundag B. Bayik Y. (2002). Serum leptin and cholesterol levels in patients with bipolar disorder. Neuropsychobiology 46, 176–179 10.1159/000067809 12566933

[B24] Avena N. M. (2007). Examining the addictive-like properties of binge eating using an animal model of sugar dependence. Exp. Clin. Psychopharmacol. 15, 481–491 10.1037/1064-1297.15.5.481 17924782

[B25] Avena N. M. Bocarsly M. E. Hoebel B. G. (2012). Animal models of sugar and fat bingeing: relationship to food addiction and increased body weight. Methods Mol. Biol. 829, 351–365 10.1007/978-1-61779-458-2_23 22231826

[B26] Avena N. M. Murray S. Gold M. S. (2013). Comparing the effects of food restriction and overeating on brain reward systems. Exp. Gerontol. 48, 1062–1067 10.1016/j.exger.2013.03.006 23535488 PMC4013785

[B27] Avena N. M. Rada P. Hoebel B. G. (2008). Evidence for sugar addiction: behavioral and neurochemical effects of intermittent, excessive sugar intake. Neurosci. Biobehav. Rev. 32, 20–39 10.1016/j.neubiorev.2007.04.019 17617461 PMC2235907

[B28] Avena N. M. Rada P. Hoebel B. G. (2009). Sugar and fat bingeing have notable differences in addictive-like behavior. J. Nutr. 139, 623–628 10.3945/jn.108.097584 19176748 PMC2714381

[B29] Barim A. O. Aydin S. Colak R. Dag E. Deniz O. Sahin I. (2009). Ghrelin, paraoxonase and arylesterase levels in depressive patients before and after citalopram treatment. Clin. Biochem. 42, 1076–1081 10.1016/j.clinbiochem.2009.02.020 19272368

[B30] Barry D. Clarke M. Petry N. M. (2009). Obesity and its relationship to addictions: is overeating a form of addictive behavior? Am. J. Addict. 18, 439–451 10.3109/10550490903205579 19874165 PMC2910406

[B31] Bast E. S. Berry E. M. (2014). Laugh away the fat? Therapeutic humor in the control of stress-induced emotional eating. Rambam Maimonides Med. J 5, e0007 10.5041/RMMJ.10141 24498514 PMC3904482

[B32] Baxter M. G. Murray E. A. (2002). The amygdala and reward. Nat. Rev. Neurosci. 3, 563–573 10.1038/nrn875 12094212

[B33] Benedict C. Hallschmid M. Hatke A. Schultes B. Fehm H. L. Born J. (2004). Intranasal insulin improves memory in humans. Psychoneuroendocrinology 29, 1326–1334 10.1016/j.psyneuen.2004.04.003 15288712

[B34] Benoit S. C. Kemp C. J. Elias C. F. Abplanalp W. Herman J. P. Migrenne S. (2009). Palmitic acid mediates hypothalamic insulin resistance by altering PKC-theta subcellular localization in rodents. J. Clin. Invest. 119, 2577–2589 10.1172/JCI36714 19726875 PMC2735917

[B35] Benton D. Donohoe R. T. (1999). The effects of nutrients on mood. Public Health Nutr. 2, 403–409 10.1017/S1368980099000555 10610080

[B36] Benton D. Griffiths R. Haller J. (1997). Thiamine supplementation mood and cognitive functioning. Psychopharmacology (Berl.) 129, 66–71 10.1007/s002130050163 9122365

[B37] Benton D. Haller J. Fordy J. (1995). Vitamin supplementation for 1 year improves mood. Neuropsychobiology 32, 98–105 10.1159/000119220 7477807

[B38] Berg K. A. Clarke W. P. Cunningham K. A. Spampinato U. (2008). Fine-tuning serotonin2c receptor function in the brain: molecular and functional implications. Neuropharmacology 55, 969–976 10.1016/j.neuropharm.2008.06.014 18602407 PMC3124806

[B39] Berner L. A. Avena N. M. Hoebel B. G. (2008). Bingeing, self-restriction, and increased body weight in rats with limited access to a sweet-fat diet. Obesity (Silver Spring) 16, 1998–2002 10.1038/oby.2008.328 19186326

[B40] Berridge K. C. (1996). Food reward: brain substrates of wanting and liking. Neurosci. Biobehav. Rev. 20, 1–25 10.1016/0149-7634(95)00033-B 8622814

[B41] Berthoud H. R. Lenard N. R. Shin A. C. (2011). Food reward, hyperphagia, and obesity. Am. J. Physiol. Regul. Integr. Comp. Physiol. 300, R1266–R1277 10.1152/ajpregu.00028.2011 21411768 PMC3119156

[B42] Bhansali P. Dunning J. Singer S. E. David L. Schmauss C. (2007). Early life stress alters adult serotonin 2C receptor pre-mRNA editing and expression of the alpha subunit of the heterotrimeric G-protein G q. J. Neurosci. 27, 1467–1473 10.1523/JNEUROSCI.4632-06.2007 17287521 PMC6673584

[B43] Black K. J. Hershey T. Koller J. M. Videen T. O. Mintun M. A. Price J. L. (2002). A possible substrate for dopamine-related changes in mood and behavior: prefrontal and limbic effects of a D3-preferring dopamine agonist. Proc. Natl. Acad. Sci. U.S.A. 99, 17113–17118 10.1073/pnas.012260599 12482941 PMC139278

[B44] Blasio A. Steardo L. Sabino V. Cottone P. (2013). Opioid system in the medial prefrontal cortex mediates binge-like eating. Addict. Biol. 19, 652–662 10.1111/adb.12033 23346966 PMC3664255

[B45] Blevins J. E. Baskin D. G. (2010). Hypothalamic-brainstem circuits controlling eating. Forum Nutr. 63, 133–140 10.1159/000264401 19955781 PMC6236679

[B46] Block J. P. He Y. Zaslavsky A. M. Ding L. Ayanian J. Z. (2009). Psychosocial stress and change in weight among US adults. Am. J. Epidemiol. 170, 181–192 10.1093/aje/kwp104 19465744 PMC2727271

[B47] Bongers P. Jansen A. Havermans R. Roefs A. Nederkoorn C. (2013). Happy eating: the underestimated role of overeating in a positive mood. Appetite 67, 74–80 10.1016/j.appet.2013.03.017 23583314

[B48] Boyle M. P. Brewer J. A. Funatsu M. Wozniak D. F. Tsien J. Z. Izumi Y. (2005). Acquired deficit of forebrain glucocorticoid receptor produces depression-like changes in adrenal axis regulation and behavior. Proc. Natl. Acad. Sci. U.S.A. 102, 473–478 10.1073/pnas.0406458102 15623560 PMC544280

[B49] Boyle M. P. Kolber B. J. Vogt S. K. Wozniak D. F. Muglia L. J. (2006). Forebrain glucocorticoid receptors modulate anxiety-associated locomotor activation and adrenal responsiveness. J. Neurosci. 26, 1971–1978 10.1523/JNEUROSCI.2173-05.2006 16481429 PMC6674945

[B50] Brown E. S. Varghese F. P. Mcewen B. S. (2004). Association of depression with medical illness: does cortisol play a role? Biol. Psychiatry 55, 1–9 10.1016/S0006-3223(03)00473-6 14706419

[B51] Bruch H. (1985). Four Decades of Eating Disorders. New York, NY: Guilford Press

[B52] Burghardt P. R. Love T. M. Stohler C. S. Hodgkinson C. Shen P. H. Enoch M. A. (2012). Leptin regulates dopamine responses to sustained stress in humans. J. Neurosci. 32, 15369–15376 10.1523/JNEUROSCI.2521-12.2012 23115175 PMC3503485

[B53] Burns C. M. Chu H. Rueter S. M. Hutchinson L. K. Canton H. Sanders-Bush E. (1997). Regulation of serotonin-2C receptor G-protein coupling by RNA editing. Nature 387, 303–308 10.1038/387303a0 9153397

[B54] Canetti L. Bachar E. Berry E. M. (2002). Food and emotion. Behav. Processes 60, 157–164 10.1016/S0376-6357(02)00082-7 12426067

[B55] Cantello R. Aguggia M. Gilli M. Delsedime M. Chiardo Cutin I. Riccio A. (1989). Major depression in Parkinson's disease and the mood response to intravenous methylphenidate: possible role of the “hedonic” dopamine synapse. J. Neurol. Neurosurg. Psychiatry. 52, 724–731 10.1136/jnnp.52.6.724 2664088 PMC1032022

[B56] Carroll B. J. Iranmanesh A. Keenan D. M. Cassidy F. Wilson W. H. Veldhuis J. D. (2012). Pathophysiology of hypercortisolism in depression: pituitary and adrenal responses to low glucocorticoid feedback. Acta Psychiatry Scand. 125, 478–491 10.1111/j.1600-0447.2011.01821.x 22211368 PMC3893569

[B57] Cartwright F. Stritzke W. G. Durkin K. Houghton S. Burke V. Beilin L. J. (2007). Chocolate craving among children: implications for disordered eating patterns. Appetite 48, 87–95 10.1016/j.appet.2006.07.081 17074419

[B58] Cartwright M. Wardle J. Steggles N. Simon A. E. Croker H. Jarvis M. J. (2003). Stress and dietary practices in adolescents. Health Psychol. 22, 362–369 10.1037/0278-6133.22.4.362 12940392

[B59] Cawley E. I. Park S. Aan Het Rot M. Sancton K. Benkelfat C. Young S. N. (2013). Dopamine and light: dissecting effects on mood and motivational states in women with subsyndromal seasonal affective disorder. J. Psychiatry Neurosci. 38, 388–397 10.1503/jpn.120181 23735584 PMC3819153

[B60] Chapman C. D. Frey W. H. 2nd. Craft S. Danielyan L. Hallschmid M. Schioth H. B. (2013). Intranasal treatment of central nervous system dysfunction in humans. Pharm. Res. 30, 2475–2484 10.1007/s11095-012-0915-1 23135822 PMC3761088

[B61] Christensen L. Brooks A. (2006). Changing food preference as a function of mood. J. Psychol. 140, 293–306 10.3200/JRLP.140.4.293-306 16967737

[B62] Christiansen A. M. Herman J. P. Ulrich-Lai Y. M. (2011). Regulatory interactions of stress and reward on rat forebrain opioidergic and GABAergic circuitry. Stress 14, 205–215 10.3109/10253890.2010.531331 21291318 PMC3140340

[B63] Chuang J. C. Perello M. Sakata I. Osborne-Lawrence S. Savitt J. M. Lutter M. (2011). Ghrelin mediates stress-induced food-reward behavior in mice. J. Clin. Invest. 121, 2684–2692 10.1172/JCI57660 21701068 PMC3223843

[B64] Cizza G. Romagni P. Lotsikas A. Lam G. Rosenthal N. E. Chrousos G. P. (2005). Plasma leptin in men and women with seasonal affective disorder and in healthy matched controls. Horm. Metab. Res. 37, 45–48 10.1055/s-2005-861033 15702439

[B65] Clark S. M. Saules K. K. (2013). Validation of the Yale Food Addiction Scale among a weight-loss surgery population. Eat. Behav. 14, 216–219 10.1016/j.eatbeh.2013.01.002 23557824

[B66] Clifton P. G. Rusk I. N. Cooper S. J. (1991). Effects of dopamine D1 and dopamine D2 antagonists on the free feeding and drinking patterns of rats. Behav. Neurosci. 105, 272–281 10.1037/0735-7044.105.2.272 1675060

[B67] Cnop M. Havel P. J. Utzschneider K. M. Carr D. B. Sinha M. K. Boyko E. J. (2003). Relationship of adiponectin to body fat distribution, insulin sensitivity and plasma lipoproteins: evidence for independent roles of age and sex. Diabetologia 46, 459–469 10.1007/s00125-003-1074-z 12687327

[B68] Collin M. Hakansson-Ovesjo M. L. Misane I. Ogren S. O. Meister B. (2000). Decreased 5-HT transporter mRNA in neurons of the dorsal raphe nucleus and behavioral depression in the obese leptin-deficient ob/ob mouse. Brain Res. Mol. Brain Res. 81, 51–61 10.1016/S0169-328X(00)00167-4 11000478

[B69] Collura L. A. Hoffman J. B. Wilson M. E. (2009). Administration of human leptin differentially affects parameters of cortisol secretion in socially housed female rhesus monkeys. Endocrine 36, 530–537 10.1007/s12020-009-9250-7 19856138

[B70] Conklin S. M. Harris J. I. Manuck S. B. Yao J. K. Hibbeln J. R. Muldoon M. F. (2007). Serum omega-3 fatty acids are associated with variation in mood, personality and behavior in hypercholesterolemic community volunteers. Psychiatry Res. 152, 1–10 10.1016/j.psychres.2006.10.006 17383013

[B71] Coppen A. Bolander-Gouaille C. (2005). Treatment of depression: time to consider folic acid and vitamin B12. J. Psychopharmacol. 19, 59–65 10.1177/0269881105048899 15671130

[B72] Cottone P. Sabino V. Steardo L. Zorrilla E. P. (2009). Consummatory, anxiety-related and metabolic adaptations in female rats with alternating access to preferred food. Psychoneuroendocrinology 34, 38–49 10.1016/j.psyneuen.2008.08.010 18842344 PMC3224792

[B73] Cummings D. E. Overduin J. (2007). Gastrointestinal regulation of food intake. J. Clin. Invest. 117, 13–23 10.1172/JCI30227 17200702 PMC1716217

[B74] Dagher A. (2013). Is obesity caused by food addiction? Can J. Diabetes 37(Suppl. 2), S235–S236 10.1016/j.jcjd.2013.03.122

[B75] Dallman M. F. (2010). Stress-induced obesity and the emotional nervous system. Trends Endocrinol. Metab. 21, 159–165 10.1016/j.tem.2009.10.004 19926299 PMC2831158

[B76] Dallman M. F. Pecoraro N. Akana S. F. La Fleur S. E. Gomez F. Houshyar H. (2003). Chronic stress and obesity: a new view of “comfort food.” Proc. Natl. Acad. Sci. U.S.A. 100, 11696–11701 10.1073/pnas.1934666100 12975524 PMC208820

[B77] Dallman M. F. Pecoraro N. C. La Fleur S. E. (2005). Chronic stress and comfort foods: self-medication and abdominal obesity. Brain Behav. Immun. 19, 275–280 10.1016/j.bbi.2004.11.004 15944067

[B78] Dallman M. F. Pecoraro N. C. La Fleur S. E. Warne J. P. Ginsberg A. B. Akana S. F. (2006). Glucocorticoids, chronic stress, and obesity. Prog. Brain Res. 153, 75–105 10.1016/S0079-6123(06)53004-3 16876569

[B79] Date Y. Murakami N. Toshinai K. Matsukura S. Niijima A. Matsuo H. (2002). The role of the gastric afferent vagal nerve in ghrelin-induced feeding and growth hormone secretion in rats. Gastroenterology 123, 1120–1128 10.1053/gast.2002.35954 12360474

[B80] Daubert E. A. Condron B. G. (2010). Serotonin: a regulator of neuronal morphology and circuitry. Trends Neurosci. 33, 424–434 10.1016/j.tins.2010.05.005 20561690 PMC2929308

[B81] Davis C. (2013). From Passive Overeating to “Food Addiction”: a Spectrum of Compulsion and Severity. ISRN Obes. 2013, 435027 10.1155/2013/435027 24555143 PMC3901973

[B82] Davis C. Curtis C. Levitan R. D. Carter J. C. Kaplan A. S. Kennedy J. L. (2011). Evidence that ‘food addiction’ is a valid phenotype of obesity. Appetite 57, 711–717 10.1016/j.appet.2011.08.017 21907742

[B83] Davis C. Levitan R. D. Kaplan A. S. Kennedy J. L. Carter J. C. (2014). Food cravings, appetite, and snack-food consumption in response to a psychomotor stimulant drug: the moderating effect of “food-addiction.” Front. Psychol. 5:403 10.3389/fpsyg.2014.00403 24847301 PMC4021123

[B84] Davis C. A. Levitan R. D. Reid C. Carter J. C. Kaplan A. S. Patte K. A. (2009). Dopamine for “wanting” and opioids for “liking”: a comparison of obese adults with and without binge eating. Obesity (Silver Spring) 17, 1220–1225 10.1038/oby.2009.52 19282821

[B85] De Groot M. Anderson R. Freedland K. E. Clouse R. E. Lustman P. J. (2001). Association of depression and diabetes complications: a meta-analysis. Psychosom. Med. 63, 619–630 10.1097/00006842-200107000-00015 11485116

[B86] De Souza C. T. Araujo E. P. Bordin S. Ashimine R. Zollner R. L. Boschero A. C. (2005). Consumption of a fat-rich diet activates a proinflammatory response and induces insulin resistance in the hypothalamus. Endocrinology 146, 4192–4199 10.1210/en.2004-1520 16002529

[B87] De Wit L. Luppino F. Van Straten A. Penninx B. Zitman F. Cuijpers P. (2010). Depression and obesity: a meta-analysis of community-based studies. Psychiatry Res. 178, 230–235 10.1016/j.psychres.2009.04.015 20462641

[B88] Degawa-Yamauchi M. Bovenkerk J. E. Juliar B. E. Watson W. Kerr K. Jones R. (2003). Serum resistin (FIZZ3) protein is increased in obese humans. J. Clin. Endocrinol. Metab. 88, 5452–5455 10.1210/jc.2002-021808 14602788

[B89] Delporte M. L. El Mkadem S. A. Quisquater M. Brichard S. M. (2004). Leptin treatment markedly increased plasma adiponectin but barely decreased plasma resistin of ob/ob mice. Am. J. Physiol. Endocrinol. Metab. 287, E446–E453 10.1152/ajpendo.00488.2003 15126241

[B90] Despres J. P. Lemieux I. Almeras N. (2006). Contribution of CB1 blockade to the management of high-risk abdominal obesity. Int. J. Obes. (Lond.) 30(Suppl. 1), S44–S52 10.1038/sj.ijo.0803278 16570106

[B91] Dess N. K. Edelheit D. (1998). The bitter with the sweet: the taste/stress/temperament nexus. Biol. Psychol. 48, 103–119 10.1016/S0301-0511(98)00014-3 9700013

[B92] Deuschle M. Blum W. F. Englaro P. Schweiger U. Weber B. Pflaum C. D. (1996). Plasma leptin in depressed patients and healthy controls. Horm. Metab. Res. 28, 714–717 10.1055/s-2007-979885 9013749

[B93] Dickson S. L. Egecioglu E. Landgren S. Skibicka K. P. Engel J. A. Jerlhag E. (2011). The role of the central ghrelin system in reward from food and chemical drugs. Mol. Cell. Endocrinol. 340, 80–87 10.1016/j.mce.2011.02.017 21354264

[B94] Diehl D. J. Gershon S. (1992). The role of dopamine in mood disorders. Compr. Psychiatry 33, 115–120 10.1016/0010-440X(92)90007-D 1347497

[B95] Dileone R. J. Taylor J. R. Picciotto M. R. (2012). The drive to eat: comparisons and distinctions between mechanisms of food reward and drug addiction. Nat. Neurosci. 15, 1330–1335 10.1038/nn.3202 23007187 PMC3570269

[B96] Diz-Chaves Y. (2011). Ghrelin, appetite regulation, and food reward: interaction with chronic stress. Int. J. Pept. 2011:898450 10.1155/2011/898450 21949667 PMC3178114

[B97] Dong C. Sanchez L. E. Price R. A. (2004). Relationship of obesity to depression: a family-based study. Int. J. Obes. Relat. Metab. Disord. 28, 790–795 10.1038/sj.ijo.0802626 15024401

[B98] Doumatey A. P. Bentley A. R. Zhou J. Huang H. Adeyemo A. Rotimi C. N. (2012). Paradoxical hyperadiponectinemia is associated with the Metabolically Healthy Obese (MHO) PHENOTYPE in African Americans. J. Endocrinol. Metab. 2, 51–65 10.4021/jem95w 23293696 PMC3534968

[B99] Duarte C. S. Sourander A. Nikolakaros G. Pihlajamaki H. Helenius H. Piha J. (2010). Child mental health problems and obesity in early adulthood. J. Pediatr. 156, 93–97 10.1016/j.jpeds.2009.06.066 19783001 PMC3586427

[B100] Egecioglu E. Skibicka K. P. Hansson C. Alvarez-Crespo M. Friberg P. A. Jerlhag E. (2011). Hedonic and incentive signals for body weight control. Rev. Endocr. Metab. Disord. 12, 141–151 10.1007/s11154-011-9166-4 21340584 PMC3145094

[B101] Eissa Ahmed A. A. Al-Rasheed N. M. Al-Rasheed N. M. (2009). Antidepressant-like effects of rosiglitazone, a PPARgamma agonist, in the rat forced swim and mouse tail suspension tests. Behav. Pharmacol. 20, 635–642 10.1097/FBP.0b013e328331b9bf 19745723

[B102] Elliott R. Friston K. J. Dolan R. J. (2000). Dissociable neural responses in human reward systems. J. Neurosci. 20, 6159–6165 10934265 10.1523/JNEUROSCI.20-16-06159.2000 PMC6772605

[B103] Englander M. T. Dulawa S. C. Bhansali P. Schmauss C. (2005). How stress and fluoxetine modulate serotonin 2C receptor pre-mRNA editing. J. Neurosci. 25, 648–651 10.1523/JNEUROSCI.3895-04.2005 15659601 PMC6725319

[B104] Epel E. Jimenez S. Brownell K. Stroud L. Stoney C. Niaura R. (2004). Are stress eaters at risk for the metabolic syndrome? Ann. N. Y. Acad. Sci. 1032, 208–210 10.1196/annals.1314.022 15677412

[B105] Esel E. Ozsoy S. Tutus A. Sofuoglu S. Kartalci S. Bayram F. (2005). Effects of antidepressant treatment and of gender on serum leptin levels in patients with major depression. Prog. Neuropsychopharmacol. Biol. Psychiatry 29, 565–570 10.1016/j.pnpbp.2005.01.009 15866359

[B106] Estruch R. Salas-Salvado J. (2013). Towards an even healthier Mediterranean diet. Nutr. Metab. Cardiovasc. Dis. 23, 1163–1166 10.1016/j.numecd.2013.09.003 24263037

[B107] Fedoroff I. Polivy J. Herman C. P. (2003). The specificity of restrained versus unrestrained eaters' responses to food cues: general desire to eat, or craving for the cued food? Appetite 41, 7–13 10.1016/S0195-6663(03)00026-6 12880616

[B108] Figueiredo H. F. Bodie B. L. Tauchi M. Dolgas C. M. Herman J. P. (2003). Stress integration after acute and chronic predator stress: differential activation of central stress circuitry and sensitization of the hypothalamo-pituitary-adrenocortical axis. Endocrinology 144, 5249–5258 10.1210/en.2003-0713 12960031

[B109] Finger B. C. Dinan T. G. Cryan J. F. (2010). Leptin-deficient mice retain normal appetitive spatial learning yet exhibit marked increases in anxiety-related behaviours. Psychopharmacology (Berl.) 210, 559–568 10.1007/s00213-010-1858-z 20422404

[B110] Finger B. C. Dinan T. G. Cryan J. F. (2011). High-fat diet selectively protects against the effects of chronic social stress in the mouse. Neuroscience 192, 351–360 10.1016/j.neuroscience.2011.06.072 21742017

[B111] Finger B. C. Dinan T. G. Cryan J. F. (2012). The temporal impact of chronic intermittent psychosocial stress on high-fat diet-induced alterations in body weight. Psychoneuroendocrinology 37, 729–741 10.1016/j.psyneuen.2011.06.015 21783325

[B112] Finlayson G. King N. Blundell J. E. (2007). Liking vs. wanting food: importance for human appetite control and weight regulation. Neurosci. Biobehav. Rev. 31, 987–1002 10.1016/j.neubiorev.2007.03.004 17559933

[B113] Fisher W. W. Thompson R. H. Piazza C. C. Crosland K. Gotjen D. (1997). On the relative reinforcing effects of choice and differential consequences. J. Appl. Behav. Anal. 30, 423–438 10.1901/jaba.1997.30-423 9316257 PMC1284060

[B114] Fletcher B. C. Pine K. J. Woodbridge Z. Nash A. (2007). How visual images of chocolate affect the craving and guilt of female dieters. Appetite 48, 211–217 10.1016/j.appet.2006.09.002 17055111

[B115] Fochtmann L. Fink M. (1992). Role of dopamine in mood disorders. Compr. Psychiatry 33, 417–418 10.1016/0010-440X(92)90065-X 1298235

[B116] Fortuna J. L. (2012). The obesity epidemic and food addiction: clinical similarities to drug dependence. J. Psychoactive Drugs 44, 56–63 10.1080/02791072.2012.662092 22641965

[B117] Fulton S. (2010). Appetite and reward. Front. Neuroendocrinol. 31, 85–103 10.1016/j.yfrne.2009.10.003 19822167

[B118] Fulton S. Pissios P. Manchon R. P. Stiles L. Frank L. Pothos E. N. (2006). Leptin regulation of the mesoaccumbens dopamine pathway. Neuron 51, 811–822 10.1016/j.neuron.2006.09.006 16982425

[B119] Garthwaite T. L. Martinson D. R. Tseng L. F. Hagen T. C. Menahan L. A. (1980). A longitudinal hormonal profile of the genetically obese mouse. Endocrinology 107, 671–676 10.1210/endo-107-3-671 6249569

[B120] Ge J. F. Qi C. C. Zhou J. N. (2013). Imbalance of leptin pathway and hypothalamus synaptic plasticity markers are associated with stress-induced depression in rats. Behav. Brain Res. 249, 38–43 10.1016/j.bbr.2013.04.020 23619134

[B121] Gearhardt A. N. Corbin W. R. Brownell K. D. (2009). Preliminary validation of the Yale Food Addiction Scale. Appetite 52, 430–436 10.1016/j.appet.2008.12.003 19121351

[B122] Gearhardt A. N. Yokum S. Orr P. T. Stice E. Corbin W. R. Brownell K. D. (2011). Neural correlates of food addiction. Arch. Gen. Psychiatry 68, 808–816 10.1001/archgenpsychiatry.2011.32 21464344 PMC3980851

[B123] Gecici O. Kuloglu M. Atmaca M. Tezcan A. E. Tunckol H. Emul H. M. (2005). High serum leptin levels in depressive disorders with atypical features. Psychiatry Clin. Neurosci. 59, 736–738 10.1111/j.1440-1819.2005.01445.x 16401252

[B124] Gibson C. D. Carnell S. Ochner C. N. Geliebter A. (2010). Neuroimaging, gut peptides and obesity: novel studies of the neurobiology of appetite. J. Neuroendocrinol. 22, 833–845 10.1111/j.1365-2826.2010.02025.x 20553371 PMC3121301

[B125] Gibson E. L. (2006). Emotional influences on food choice: sensory, physiological and psychological pathways. Physiol. Behav. 89, 53–61 10.1016/j.physbeh.2006.01.024 16545403

[B126] Gibson E. L. (2012). The psychobiology of comfort eating: implications for neuropharmacological interventions. Behav. Pharmacol. 23, 442–460 10.1097/FBP.0b013e328357bd4e 22854304

[B127] Go A. S. Mozaffarian D. Roger V. L. Benjamin E. J. Berry J. D. Blaha M. J. (2014). Heart disease and stroke statistics–2014 update: a report from the American Heart Association. Circulation 129, e28–e292 10.1161/01.cir.0000442015.53336.12 24352519 PMC5408159

[B128] Go A. S. Mozaffarian D. Roger V. L. Benjamin E. J. Berry J. D. Borden W. B. (2013a). Executive summary: heart disease and stroke statistics–2013 update: a report from the American Heart Association. Circulation 127, 143–152 10.1161/CIR.0b013e318282ab8f 23283859

[B129] Go A. S. Mozaffarian D. Roger V. L. Benjamin E. J. Berry J. D. Borden W. B. (2013b). Heart disease and stroke statistics–2013 update: a report from the American Heart Association. Circulation 127, e6–e245 10.1161/CIR.0b013e31828124ad 23239837 PMC5408511

[B130] Gonder-Frederick La C. D. Bobbitt S. A. Pennebaker J. W. (1989). Mood changes associated with blood glucose fluctuations in insulin-dependent diabetes mellitus. Health Psychol. 8, 45–59 10.1037/0278-6133.8.1.45 2707223

[B131] Granados K. Stephens B. R. Malin S. K. Zderic T. W. Hamilton M. T. Braun B. (2012). Appetite regulation in response to sitting and energy imbalance. Appl. Physiol. Nutr. Metab. 37, 323–333 10.1139/h2012-002 22462636

[B132] Greeno C. G. Wing R. R. (1994). Stress-induced eating. Psychol. Bull. 115, 444–464 10.1037/0033-2909.115.3.444 8016287

[B133] Grillo C. A. Piroli G. G. Kaigler K. F. Wilson S. P. Wilson M. A. Reagan L. P. (2011). Downregulation of hypothalamic insulin receptor expression elicits depressive-like behaviors in rats. Behav. Brain Res. 222, 230–235 10.1016/j.bbr.2011.03.052 21458499 PMC3774048

[B134] Grosso G. Galvano F. Marventano S. Malaguarnera M. Bucolo C. Drago F. (2014). Omega-3 fatty acids and depression: scientific evidence and biological mechanisms. Oxid. Med. Cell. Longev. 2014, 313570 10.1155/2014/313570 24757497 PMC3976923

[B135] Guo M. Huang T. Y. Garza J. C. Chua S. C. Lu X. Y. (2013). Selective deletion of leptin receptors in adult hippocampus induces depression-related behaviours. Int. J. Neuropsychopharmacol. 16, 857–867 10.1017/S1461145712000703 22932068 PMC3612133

[B136] Guo M. Lu Y. Garza J. C. Li Y. Chua S. C. Zhang W. (2012). Forebrain glutamatergic neurons mediate leptin action on depression-like behaviors and synaptic depression. Transl. Psychiatry 2, e83 10.1038/tp.2012.9 22408745 PMC3298113

[B137] Gurevich I. Englander M. T. Adlersberg M. Siegal N. B. Schmauss C. (2002). Modulation of serotonin 2C receptor editing by sustained changes in serotonergic neurotransmission. J. Neurosci. 22, 10529–10532 12486144 10.1523/JNEUROSCI.22-24-10529.2002 PMC6758441

[B138] Gustafson T. A. Moodie S. A. Lavan B. E. (1999). The insulin receptor and metabolic signaling. Rev. Physiol. Biochem. Pharmacol. 137, 71–190 10207305 10.1007/3-540-65362-7_5

[B139] Hamer M. Batty G. D. Kivimaki M. (2012). Risk of future depression in people who are obese but metabolically healthy: the English longitudinal study of ageing. Mol. Psychiatry 17, 940–945 10.1038/mp.2012.30 22525487 PMC3428506

[B140] Hammack S. E. Roman C. W. Lezak K. R. Kocho-Shellenberg M. Grimmig B. Falls W. A. (2010). Roles for pituitary adenylate cyclase-activating peptide (PACAP) expression and signaling in the bed nucleus of the stria terminalis (BNST) in mediating the behavioral consequences of chronic stress. J. Mol. Neurosci. 42, 327–340 10.1007/s12031-010-9364-7 20405238 PMC2955825

[B141] Hanley A. J. Bowden D. Wagenknecht L. E. Balasubramanyam A. Langfeld C. Saad M. F. (2007). Associations of adiponectin with body fat distribution and insulin sensitivity in nondiabetic Hispanics and African-Americans. J. Clin. Endocrinol. Metab. 92, 2665–2671 10.1210/jc.2006-2614 17426091

[B142] Hayes D. J. Greenshaw A. J. (2011). 5-HT receptors and reward-related behaviour: a review. Neurosci. Biobehav. Rev. 35, 1419–1449 10.1016/j.neubiorev.2011.03.005 21402098

[B143] Herman C. P. Mack D. (1975). Restrained and unrestrained eating. J. Pers. 43, 647–660 10.1111/j.1467-6494.1975.tb00727.x 1206453

[B144] Heyne A. Kiesselbach C. Sahun I. Mcdonald J. Gaiffi M. Dierssen M. (2009). An animal model of compulsive food-taking behaviour. Addict. Biol. 14, 373–383 10.1111/j.1369-1600.2009.00175.x 19740365

[B145] Higgins G. A. Fletcher P. J. (2003). Serotonin and drug reward: focus on 5-HT2C receptors. Eur. J. Pharmacol. 480, 151–162 10.1016/j.ejphar.2003.08.102 14623358

[B146] Hnasko T. S. Szczypka M. S. Alaynick W. A. During M. J. Palmiter R. D. (2004). A role for dopamine in feeding responses produced by orexigenic agents. Brain Res. 1023, 309–318 10.1016/j.brainres.2004.07.051 15374756

[B147] Holsboer F. (2000). The corticosteroid receptor hypothesis of depression. Neuropsychopharmacology 23, 477–501 10.1016/S0893-133X(00)00159-7 11027914

[B148] Hommel J. D. Trinko R. Sears R. M. Georgescu D. Liu Z. W. Gao X. B. (2006). Leptin receptor signaling in midbrain dopamine neurons regulates feeding. Neuron 51, 801–810 10.1016/j.neuron.2006.08.023 16982424

[B149] Hone-Blanchet A. Fecteau S. (2014). Overlap of food addiction and substance use disorders definitions: analysis of animal and human studies. Neuropharmacology 85C, 81–90 10.1016/j.neuropharm.2014.05.019 24863044

[B150] Hryhorczuk C. Sharma S. Fulton S. E. (2013). Metabolic disturbances connecting obesity and depression. Front. Neurosci. 7:177 10.3389/fnins.2013.00177 24109426 PMC3791387

[B151] Huether G. Zhou D. Schmidt S. Wiltfang J. Ruther E. (1997). Long-term food restriction down-regulates the density of serotonin transporters in the rat frontal cortex. Biol. Psychiatry 41, 1174–1180 10.1016/S0006-3223(96)00265-X 9171908

[B152] Iemolo A. Valenza M. Tozier L. Knapp C. M. Kornetsky C. Steardo L. (2012). Withdrawal from chronic, intermittent access to a highly palatable food induces depressive-like behavior in compulsive eating rats. Behav. Pharmacol. 23, 593–602 10.1097/FBP.0b013e328357697f 22854309 PMC3934429

[B153] Iwamoto K. Kato T. (2003). RNA editing of serotonin 2C receptor in human postmortem brains of major mental disorders. Neurosci. Lett. 346, 169–172 10.1016/S0304-3940(03)00608-6 12853111

[B154] Iwamoto K. Nakatani N. Bundo M. Yoshikawa T. Kato T. (2005). Altered RNA editing of serotonin 2C receptor in a rat model of depression. Neurosci. Res. 53, 69–76 10.1016/j.neures.2005.06.001 16005997

[B155] Jauch-Chara K. Oltmanns K. M. (2014). Obesity–A neuropsychological disease? Systematic review and neuropsychological model. Prog. Neurobiol. 114C, 84–101 10.1016/j.pneurobio.2013.12.001 24394671

[B156] Jeong H. G. Min B. J. Lim S. Kim T. H. Lee J. J. Park J. H. (2012). Plasma adiponectin elevation in elderly individuals with subsyndromal depression. Psychoneuroendocrinology 37, 948–955 10.1016/j.psyneuen.2011.11.002 22130479

[B157] Jow G. M. Yang T. T. Chen C. L. (2006). Leptin and cholesterol levels are low in major depressive disorder, but high in schizophrenia. J. Affect. Disord. 90, 21–27 10.1016/j.jad.2005.09.015 16324751

[B158] Juruena M. F. Cleare A. J. (2007). [Overlap between atypical depression, seasonal affective disorder and chronic fatigue syndrome]. Rev. Bras. Psiquiatr. 29(Suppl. 1), S19–S26 10.1590/S1516-44462007000500005 17546343

[B159] Kan C. Silva N. Golden S. H. Rajala U. Timonen M. Stahl D. (2013). A systematic review and meta-analysis of the association between depression and insulin resistance. Diabetes Care 36, 480–489 10.2337/dc12-1442 23349152 PMC3554272

[B160] Kang J. X. Gleason E. D. (2013). Omega-3 Fatty acids and hippocampal neurogenesis in depression. CNS Neurol. Disord. Drug Targets 12, 460–465 10.2174/1871527311312040004 23574158

[B161] Kaplan H. I. Kaplan H. S. (1957). The psychosomatic concept of obesity. J. Nerv. Ment. Dis. 125, 181–201 13481715 10.1097/00005053-195704000-00004

[B162] Karra E. O'daly O. G. Choudhury A. I. Yousseif A. Millership S. Neary M. T. (2013). A link between FTO, ghrelin, and impaired brain food-cue responsivity. J. Clin. Invest. 123, 3539–3551 10.1172/JCI44403 23867619 PMC3726147

[B163] Kawahara Y. Grimberg A. Teegarden S. Mombereau C. Liu S. Bale T. L. (2008). Dysregulated editing of serotonin 2C receptor mRNAs results in energy dissipation and loss of fat mass. J. Neurosci. 28, 12834–12844 10.1523/JNEUROSCI.3896-08.2008 19036977 PMC2615198

[B164] Kemp A. H. Quintana D. S. Felmingham K. L. Matthews S. Jelinek H. F. (2012). Depression, comorbid anxiety disorders, and heart rate variability in physically healthy, unmedicated patients: implications for cardiovascular risk. PLoS ONE 7:e30777 10.1371/journal.pone.0030777 22355326 PMC3280258

[B165] Kennedy L. Bittel D. C. Kibiryeva N. Kalra S. P. Torto R. Butler M. G. (2006). Circulating adiponectin levels, body composition and obesity-related variables in Prader-Willi syndrome: comparison with obese subjects. Int. J. Obes. (Lond.) 30, 382–387 10.1038/sj.ijo.0803115 16231029 PMC6704478

[B166] Kiefer F. Jahn H. Kellner M. Naber D. Wiedemann K. (2001). Leptin as a possible modulator of craving for alcohol. Arch. Gen. Psychiatry 58, 509–510 10.1001/archpsyc.58.5.509 11343532

[B167] Kim B. Feldman E. L. (2012). Insulin resistance in the nervous system. Trends Endocrinol. Metab. 23, 133–141 10.1016/j.tem.2011.12.004 22245457 PMC3392648

[B168] Kleinridders A. Schenten D. Konner A. C. Belgardt B. F. Mauer J. Okamura T. (2009). MyD88 signaling in the CNS is required for development of fatty acid-induced leptin resistance and diet-induced obesity. Cell Metab. 10, 249–259 10.1016/j.cmet.2009.08.013 19808018 PMC3898351

[B169] Kloiber S. Ising M. Reppermund S. Horstmann S. Dose T. Majer M. (2007). Overweight and obesity affect treatment response in major depression. Biol. Psychiatry 62, 321–326 10.1016/j.biopsych.2006.10.001 17241618

[B170] Kluge M. Schussler P. Dresler M. Schmidt D. Yassouridis A. Uhr M. (2011). Effects of ghrelin on psychopathology, sleep and secretion of cortisol and growth hormone in patients with major depression. J. Psychiatry Res. 45, 421–426 10.1016/j.jpsychires.2010.09.002 20888580

[B171] Kluge M. Schussler P. Schmid D. Uhr M. Kleyer S. Yassouridis A. (2009). Ghrelin plasma levels are not altered in major depression. Neuropsychobiology 59, 199–204 10.1159/000223731 19521111

[B172] Kojima M. Hosoda H. Date Y. Nakazato M. Matsuo H. Kangawa K. (1999). Ghrelin is a growth-hormone-releasing acylated peptide from stomach. Nature 402, 656–660 10.1038/45230 10604470

[B173] Kojima M. Hosoda H. Kangawa K. (2004). Clinical endocrinology and metabolism. Ghrelin, a novel growth-hormone-releasing and appetite-stimulating peptide from stomach. Best Pract. Res. Clin. Endocrinol. Metab. 18, 517–530 10.1016/j.beem.2004.07.001 15533773

[B174] Komorowski J. Jankiewicz-Wika J. Stepien H. (2000). Effects of Gn-RH, TRH, and CRF administration on plasma leptin levels in lean and obese women. Neuropeptides 34, 89–97 10.1054/npep.2000.0799 10985925

[B175] Koob G. F. Volkow N. D. (2010). Neurocircuitry of addiction. Neuropsychopharmacology 35, 217–238 10.1038/npp.2009.110 19710631 PMC2805560

[B176] Koponen H. Jokelainen J. Keinanen-Kiukaanniemi S. Kumpusalo E. Vanhala M. (2008). Metabolic syndrome predisposes to depressive symptoms: a population-based 7-year follow-up study. J. Clin. Psychiatry 69, 178–182 10.4088/JCP.v69n0202 18232723

[B177] Kraus D. Fasshauer M. Ott V. Meier B. Jost M. Klein H. H. (2002). Leptin secretion and negative autocrine crosstalk with insulin in brown adipocytes. J. Endocrinol. 175, 185–191 10.1677/joe.0.1750185 12379502

[B178] Kraus T. Haack M. Schuld A. Hinze-Selch D. Pollmacher T. (2001). Low leptin levels but normal body mass indices in patients with depression or schizophrenia. Neuroendocrinology 73, 243–247 10.1159/000054641 11340338

[B179] Krishnan V. Nestler E. J. (2008). The molecular neurobiology of depression. Nature 455, 894–902 10.1038/nature07455 18923511 PMC2721780

[B180] Krsek M. Silha J. V. Jezkova J. Hana V. Marek J. Weiss V. (2004). Adipokine levels in Cushing's syndrome; elevated resistin levels in female patients with Cushing's syndrome. Clin. Endocrinol. (Oxf.) 60, 350–357 10.1111/j.1365-2265.2003.01987.x 15009001

[B181] Kumar J. Chuang J. C. Na E. S. Kuperman A. Gillman A. G. Mukherjee S. (2013). Differential effects of chronic social stress and fluoxetine on meal patterns in mice. Appetite 64, 81–88 10.1016/j.appet.2012.12.023 23318656 PMC3606634

[B182] Kyrou I. Tsigos C. (2009). Stress hormones: physiological stress and regulation of metabolism. Curr. Opin. Pharmacol. 9, 787–793 10.1016/j.coph.2009.08.007 19758844

[B183] La Fleur S. E. Manalo S. L. Roy M. Houshyar H. Dallman M. F. (2005). Hepatic vagotomy alters limbic and hypothalamic neuropeptide responses to insulin-dependent diabetes and voluntary lard ingestion. Eur. J. Neurosci. 21, 2733–2742 10.1111/j.1460-9568.2005.04125.x 15926921

[B184] Labad J. Price J. F. Strachan M. W. Fowkes F. G. Deary I. J. Seckl J. R. (2012). Leptin levels and depressive symptoms in people with type 2 diabetes: the edinburgh type 2 diabetes study. Psychosom. Med. 74, 39–45 10.1097/PSY.0b013e31823ba8af 22210236

[B185] Labad J. Price J. F. Strachan M. W. Fowkes F. G. Ding J. Deary I. J. (2010). Symptoms of depression but not anxiety are associated with central obesity and cardiovascular disease in people with type 2 diabetes: the Edinburgh Type 2 Diabetes Study. Diabetologia 53, 467–471 10.1007/s00125-009-1628-9 20012009 PMC3977034

[B186] Lawson E. A. Miller K. K. Blum J. I. Meenaghan E. Misra M. Eddy K. T. (2012). Leptin levels are associated with decreased depressive symptoms in women across the weight spectrum, independent of body fat. Clin. Endocrinol. (Oxf.) 76, 520–525 10.1111/j.1365-2265.2011.04182.x 21781144 PMC3296868

[B187] Lehto S. M. Huotari A. Niskanen L. Tolmunen T. Koivumaa-Honkanen H. Honkalampi K. (2010). Serum adiponectin and resistin levels in major depressive disorder. Acta Psychiatry Scand. 121, 209–215 10.1111/j.1600-0447.2009.01463.x 19694629

[B188] Leinninger G. M. Jo Y. H. Leshan R. L. Louis G. W. Yang H. Barrera J. G. et al. Myers M. G. Jr. (2009). Leptin acts via leptin receptor-expressing lateral hypothalamic neurons to modulate the mesolimbic dopamine system and suppress feeding. Cell Metab. 10, 89–98 10.1016/j.cmet.2009.06.011 19656487 PMC2723060

[B189] Leo R. Di Lorenzo G. Tesauro M. Cola C. Fortuna E. Zanasi M. (2006). Decreased plasma adiponectin concentration in major depression. Neurosci. Lett. 407, 211–213 10.1016/j.neulet.2006.08.043 16973279

[B190] Lin P. Y. Su K. P. (2007). A meta-analytic review of double-blind, placebo-controlled trials of antidepressant efficacy of omega-3 fatty acids. J. Clin. Psychiatry 68, 1056–1061 10.4088/JCP.v68n0712 17685742

[B191] Liu J. Garza J. C. Bronner J. Kim C. S. Zhang W. Lu X. Y. (2010). Acute administration of leptin produces anxiolytic-like effects: a comparison with fluoxetine. Psychopharmacology (Berl.) 207, 535–545 10.1007/s00213-009-1684-3 19823809 PMC4057895

[B192] Liu J. Guo M. Zhang D. Cheng S. Y. Liu M. Ding J. (2012). Adiponectin is critical in determining susceptibility to depressive behaviors and has antidepressant-like activity. Proc. Natl. Acad. Sci. U.S.A. 109, 12248–12253 10.1073/pnas.1202835109 22778410 PMC3409774

[B193] Liu J. Perez S. M. Zhang W. Lodge D. J. Lu X. Y. (2011). Selective deletion of the leptin receptor in dopamine neurons produces anxiogenic-like behavior and increases dopaminergic activity in amygdala. Mol. Psychiatry 16, 1024–1038 10.1038/mp.2011.36 21483433 PMC3432580

[B194] Lombard C. B. (2000). What is the role of food in preventing depression and improving mood, performance and cognitive function? Med. J. Aust. 173(Suppl.), S104–S105 11149371 10.5694/j.1326-5377.2000.tb139438.x

[B195] Lu X. Y. (2007). The leptin hypothesis of depression: a potential link between mood disorders and obesity? Curr. Opin. Pharmacol. 7, 648–652 10.1016/j.coph.2007.10.010 18032111 PMC2677994

[B196] Lu X. Y. Kim C. S. Frazer A. Zhang W. (2006). Leptin: a potential novel antidepressant. Proc. Natl. Acad. Sci. U.S.A. 103, 1593–1598 10.1073/pnas.0508901103 16423896 PMC1360555

[B197] Luppino F. S. De Wit L. M. Bouvy P. F. Stijnen T. Cuijpers P. Penninx B. W. (2010). Overweight, obesity, and depression: a systematic review and meta-analysis of longitudinal studies. Arch. Gen. Psychiatry 67, 220–229 10.1001/archgenpsychiatry.2010.2 20194822

[B198] Lutter M. Elmquist J. (2009). Depression and metabolism: linking changes in leptin and ghrelin to mood. F1000 Biol. Rep. 1:63 10.3410/B1-63 20948621 PMC2948264

[B199] Lyman B. (1982). The nutritional values and food group characteristics of foods preferred during various emotions. J. Psychol. 112, 121–127 10.1080/00223980.1982.9923544 7143268

[B200] Lyons W. E. Mamounas L. A. Ricaurte G. A. Coppola V. Reid S. W. Bora S. H. (1999). Brain-derived neurotrophic factor-deficient mice develop aggressiveness and hyperphagia in conjunction with brain serotonergic abnormalities. Proc. Natl. Acad. Sci. U.S.A. 96, 15239–15244 10.1073/pnas.96.26.15239 10611369 PMC24804

[B201] Macht M. (1999). Characteristics of eating in anger, fear, sadness and joy. Appetite 33, 129–139 10.1006/appe.1999.0236 10447985

[B202] Macht M. (2008). How emotions affect eating: a five-way model. Appetite 50, 1–11 10.1016/j.appet.2007.07.002 17707947

[B203] Macht M. Dettmer D. (2006). Everyday mood and emotions after eating a chocolate bar or an apple. Appetite 46, 332–336 10.1016/j.appet.2006.01.014 16546294

[B204] Macht M. Gerer J. Ellgring H. (2003). Emotions in overweight and normal-weight women immediately after eating foods differing in energy. Physiol. Behav. 80, 367–374 10.1016/j.physbeh.2003.08.012 14637237

[B205] Macht M. Simons G. (2000). Emotions and eating in everyday life. Appetite 35, 65–71 10.1006/appe.2000.0325 10896762

[B206] Maeda N. Takahashi M. Funahashi T. Kihara S. Nishizawa H. Kishida K. (2001). PPARgamma ligands increase expression and plasma concentrations of adiponectin, an adipose-derived protein. Diabetes 50, 2094–2099 10.2337/diabetes.50.9.2094 11522676

[B207] Maffei M. Halaas J. Ravussin E. Pratley R. E. Lee G. H. Zhang Y. (1995). Leptin levels in human and rodent: measurement of plasma leptin and ob RNA in obese and weight-reduced subjects. Nat. Med. 1, 1155–1161 10.1038/nm1195-1155 7584987

[B208] Maniam J. Morris M. J. (2010). Voluntary exercise and palatable high-fat diet both improve behavioural profile and stress responses in male rats exposed to early life stress: role of hippocampus. Psychoneuroendocrinology 35, 1553–1564 10.1016/j.psyneuen.2010.05.012 20594764

[B209] Maniam J. Morris M. J. (2012). The link between stress and feeding behaviour. Neuropharmacology 63, 97–110 10.1016/j.neuropharm.2012.04.017 22710442

[B210] Mann J. N. Thakore J. H. (1999). Melancholic depression and abdominal fat distribution: a mini-review. Stress 3, 1–15 10.3109/10253899909001108 19016189

[B211] Marijnissen R. M. Bus B. A. Holewijn S. Franke B. Purandare N. De Graaf J. (2011). Depressive symptom clusters are differentially associated with general and visceral obesity. J. Am. Geriatr. Soc. 59, 67–72 10.1111/j.1532-5415.2010.03228.x 21226677

[B212] Marks D. R. Tucker K. Cavallin M. A. Mast T. G. Fadool D. A. (2009). Awake intranasal insulin delivery modifies protein complexes and alters memory, anxiety, and olfactory behaviors. J. Neurosci. 29, 6734–6751 10.1523/JNEUROSCI.1350-09.2009 19458242 PMC2779219

[B213] Martin C. K. Mcclernon F. J. Chellino A. Correa J. B. (2011). Food Cravings: a Central Construct in Food Intake Behavior, Weight Loss, and the Neurobiology of Appetitive Behavior. New York, NY: Springer

[B214] Matheny M. Shapiro A. Tumer N. Scarpace P. J. (2011). Region-specific diet-induced and leptin-induced cellular leptin resistance includes the ventral tegmental area in rats. Neuropharmacology 60, 480–487 10.1016/j.neuropharm.2010.11.002 21059361 PMC3014426

[B215] Mcelroy S. L. Kotwal R. Malhotra S. Nelson E. B. Keck P. E. Nemeroff C. B. (2004). Are mood disorders and obesity related? A review for the mental health professional. J. Clin. Psychiatry 65, 634–651 730. 10.4088/JCP.v65n0507 15163249

[B216] Mehrabian A. (1995). Relationships among three general approaches to personality description. J. Psychol. 129, 565–581 10.1080/00223980.1995.9914929 7473304

[B217] Merali Z. Graitson S. Mackay J. C. Kent P. (2013). Stress and eating: a dual role for bombesin-like peptides. Front. Neurosci. 7:193 10.3389/fnins.2013.00193 24298233 PMC3829480

[B218] Meye F. J. Adan R. A. (2014). Feelings about food: the ventral tegmental area in food reward and emotional eating. Trends Pharmacol. Sci. 35, 31–40 10.1016/j.tips.2013.11.003 24332673

[B219] Milan G. Granzotto M. Scarda A. Calcagno A. Pagano C. Federspil G. (2002). Resistin and adiponectin expression in visceral fat of obese rats: effect of weight loss. Obes. Res. 10, 1095–1103 10.1038/oby.2002.149 12429872

[B220] Milaneschi Y. Simonsick E. M. Vogelzangs N. Strotmeyer E. S. Yaffe K. Harris T. B. (2012). Leptin, abdominal obesity, and onset of depression in older men and women. J. Clin. Psychiatry 73, 1205–1211 10.4088/JCP.11m07552 22687702 PMC3486693

[B221] Miller A. L. (2005). Epidemiology, etiology, and natural treatment of seasonal affective disorder. Altern. Med. Rev. 10, 5–13 15771558

[B222] Molenaar E. A. Massaro J. M. Jacques P. F. Pou K. M. Ellison R. C. Hoffmann U. (2009). Association of lifestyle factors with abdominal subcutaneous and visceral adiposity: the Framingham Heart Study. Diabetes Care 32, 505–510 10.2337/dc08-1382 19074991 PMC2646037

[B223] Morabito M. V. Abbas A. I. Hood J. L. Kesterson R. A. Jacobs M. M. Kump D. S. (2010). Mice with altered serotonin 2C receptor RNA editing display characteristics of Prader-Willi syndrome. Neurobiol. Dis. 39, 169–180 10.1016/j.nbd.2010.04.004 20394819 PMC2906772

[B224] Moranis A. Delpech J. C. De Smedt-Peyrusse V. Aubert A. Guesnet P. Lavialle M. (2012). Long term adequate n-3 polyunsaturated fatty acid diet protects from depressive-like behavior but not from working memory disruption and brain cytokine expression in aged mice. Brain Behav. Immun. 26, 721–731 10.1016/j.bbi.2011.11.001 22085587

[B225] Morris J. S. Dolan R. J. (2001). Involvement of human amygdala and orbitofrontal cortex in hunger-enhanced memory for food stimuli. J. Neurosci. 21, 5304–5310 11438606 10.1523/JNEUROSCI.21-14-05304.2001 PMC6762827

[B226] Morris W. N. Reilly N. P. (1987). Toward the self-regulation of mood: theory and research. Motiv. Emot. 11, 215–249 10.1007/BF01001412

[B227] Morrison J. A. Glueck C. J. Daniels S. Wang P. Stroop D. (2011). Paradoxically high adiponectin in obese 16-year-old girls protects against appearance of the metabolic syndrome and its components seven years later. J. Pediatr. 158, 208.e201–214.e201 10.1016/j.jpeds.2010.08.012 20869727 PMC3022119

[B228] Mosienko V. Bert B. Beis D. Matthes S. Fink H. Bader M. (2012). Exaggerated aggression and decreased anxiety in mice deficient in brain serotonin. Transl. Psychiatry 2, e122 10.1038/tp.2012.44 22832966 PMC3365263

[B229] Mozaffarian D. Hao T. Rimm E. B. Willett W. C. Hu F. B. (2011). Changes in diet and lifestyle and long-term weight gain in women and men. N. Engl. J. Med. 364, 2392–2404 10.1056/NEJMoa1014296 21696306 PMC3151731

[B230] Myers M. G. Jr. Heymsfield S. B. Haft C. Kahn B. B. Laughlin M. Leibel R. L. (2012). Challenges and opportunities of defining clinical leptin resistance. Cell Metab. 15, 150–156 10.1016/j.cmet.2012.01.002 22326217 PMC3281561

[B231] Nakazato M. Murakami N. Date Y. Kojima M. Matsuo H. Kangawa K. (2001). A role for ghrelin in the central regulation of feeding. Nature 409, 194–198 10.1038/35051587 11196643

[B232] Narita K. Murata T. Takahashi T. Kosaka H. Omata N. Wada Y. (2006). Plasma levels of adiponectin and tumor necrosis factor-alpha in patients with remitted major depression receiving long-term maintenance antidepressant therapy. Prog. Neuropsychopharmacol. Biol. Psychiatry 30, 1159–1162 10.1016/j.pnpbp.2006.03.030 16678955

[B233] Nazare J. A. Smith J. Borel A. L. Almeras N. Tremblay A. Bergeron J. (2013). Changes in both global diet quality and physical activity level synergistically reduce visceral adiposity in men with features of metabolic syndrome. J. Nutr. 143, 1074–1083 10.3945/jn.113.175273 23719226

[B234] Nordquist N. Oreland L. (2010). Serotonin, genetic variability, behaviour, and psychiatric disorders–a review. Ups. J. Med. Sci. 115, 2–10 10.3109/03009730903573246 20187845 PMC2853349

[B235] Novick J. S. Stewart J. W. Wisniewski S. R. Cook I. A. Manev R. Nierenberg A. A. (2005). Clinical and demographic features of atypical depression in outpatients with major depressive disorder: preliminary findings from STAR*D. J. Clin. Psychiatry 66, 1002–1011 10.4088/JCP.v66n0807 16086615

[B236] Oddy W. H. Hickling S. Smith M. A. O'sullivan T. A. Robinson M. De Klerk N. H. (2011). Dietary intake of omega-3 fatty acids and risk of depressive symptoms in adolescents. Depress. Anxiety 28, 582–588 10.1002/da.20822 21538725

[B237] Ogden C. L. Carroll M. D. Kit B. K. Flegal K. M. (2014). Prevalence of childhood and adult obesity in the United States, 2011-2012. JAMA 311, 806–814 10.1001/jama.2014.732 24570244 PMC4770258

[B238] Olaghere Da Silva U. B. Morabito M. V. Canal C. E. Airey D. C. Emeson R. B. Sanders-Bush E. (2010). Impact of RNA editing on functions of the serotonin 2C receptor *in vivo*. Front. Neurosci. 4:26 10.3389/neuro.23.001.2010 20582266 PMC2858556

[B239] Oliver G. Wardle J. (1999). Perceived effects of stress on food choice. Physiol. Behav. 66, 511–515 10.1016/S0031-9384(98)00322-9 10357442

[B240] Olsen C. M. (2011). Natural rewards, neuroplasticity, and non-drug addictions. Neuropharmacology 61, 1109–1122 10.1016/j.neuropharm.2011.03.010 21459101 PMC3139704

[B241] Olszewski P. K. Schioth H. B. Levine A. S. (2008). Ghrelin in the CNS: from hunger to a rewarding and memorable meal? Brain Res. Rev. 58, 160–170 10.1016/j.brainresrev.2008.01.004 18308399 PMC2494866

[B242] Opland D. M. Leinninger G. M. Myers M. G. Jr. (2010). Modulation of the mesolimbic dopamine system by leptin. Brain Res. 1350, 65–70 10.1016/j.brainres.2010.04.028 20417193 PMC2921997

[B243] Organization W. H. (2013). Obesity and Overweight. World Health Organization. Available online at: http://www.who.int/mediacentre/factsheets/fs311/en/index.html

[B244] Osman J. L. Sobal J. (2006). Chocolate cravings in American and Spanish individuals: biological and cultural influences. Appetite 47, 290–301 10.1016/j.appet.2006.04.008 16831486

[B245] Ottley C. (2000). Food and mood. Nurs. Stand. 15, 46–52 11971416

[B246] Ouwens M. A. Van Strien T. Van Leeuwe J. F. (2009). Possible pathways between depression, emotional and external eating. A structural equation model. Appetite 53, 245–248 10.1016/j.appet.2009.06.001 19505515

[B247] Overduin J. Figlewicz D. P. Bennett-Jay J. Kittleson S. Cummings D. E. (2012). Ghrelin increases the motivation to eat, but does not alter food palatability. Am. J. Physiol. Regul. Integr. Comp. Physiol. 303, R259–R269 10.1152/ajpregu.00488.2011 22673784 PMC3423988

[B248] Owecki M. Miczke A. Nikisch E. Pupek-Musialik D. Sowinski J. (2011). Serum resistin concentrations are higher in human obesity but independent from insulin resistance. Exp. Clin. Endocrinol. Diabetes 119, 117–121 10.1055/s-0030-1263111 20827661

[B249] Pai N. Vella S. L. Richardson K. (2014). Is food addiction a valid phenomenon through the lens of the DSM-5? Aust. N. Z. J. Psychiatry 48, 216–218 10.1177/0004867413512384 24220135

[B250] Park E. Kim J. Y. Lee J. H. Jahng J. W. (2014). Increased depression-like behaviors with dysfunctions in the stress axis and the reward center by free access to highly palatable food. Neuroscience 262, 31–39 10.1016/j.neuroscience.2013.12.054 24406442

[B251] Park Y. Kim M. Baek D. Kim S. H. (2012a). Erythrocyte n-3 polyunsaturated fatty acid and seafood intake decrease the risk of depression: case-control study in Korea. Ann. Nutr. Metab. 61, 25–31 10.1159/000339264 22776859

[B252] Park Y. Moon H. J. Kim S. H. (2012b). N-3 polyunsaturated fatty acid consumption produces neurobiological effects associated with prevention of depression in rats after the forced swimming test. J. Nutr. Biochem. 23, 924–928 10.1016/j.jnutbio.2011.04.018 21852084

[B253] Parker G. Gibson N. A. Brotchie H. Heruc G. Rees A. M. Hadzi-Pavlovic D. (2006a). Omega-3 fatty acids and mood disorders. Am. J. Psychiatry 163, 969–978 10.1176/appi.ajp.163.6.969 16741195

[B254] Parker G. Parker I. Brotchie H. (2006b). Mood state effects of chocolate. J. Affect. Disord. 92, 149–159 10.1016/j.jad.2006.02.007 16546266

[B255] Parker K. J. Schatzberg A. F. Lyons D. M. (2003). Neuroendocrine aspects of hypercortisolism in major depression. Horm. Behav. 43, 60–66 10.1016/S0018-506X(02)00016-8 12614635

[B256] Parylak S. L. Koob G. F. Zorrilla E. P. (2011). The dark side of food addiction. Physiol. Behav. 104, 149–156 10.1016/j.physbeh.2011.04.063 21557958 PMC3304465

[B257] Pasquali R. (2012). The hypothalamic-pituitary-adrenal axis and sex hormones in chronic stress and obesity: pathophysiological and clinical aspects. Ann. N. Y. Acad. Sci. 1264, 20–35 10.1111/j.1749-6632.2012.06569.x 22612409 PMC3464358

[B258] Patterson Z. R. Abizaid A. (2013). Stress induced obesity: lessons from rodent models of stress. Front. Neurosci. 7:130 10.3389/fnins.2013.00130 23898237 PMC3721047

[B259] Pawels E. K. Volterrani D. (2008). Fatty acid facts, Part I. Essential fatty acids as treatment for depression, or food for mood? Drug News Perspect. 21, 446–451 10.1358/dnp.2008.21.8.1272136 19034351

[B260] Pecoraro N. Reyes F. Gomez F. Bhargava A. Dallman M. F. (2004). Chronic stress promotes palatable feeding, which reduces signs of stress: feedforward and feedback effects of chronic stress. Endocrinology 145, 3754–3762 10.1210/en.2004-0305 15142987

[B261] Pedram P. Wadden D. Amini P. Gulliver W. Randell E. Cahill F. (2013). Food addiction: its prevalence and significant association with obesity in the general population. PLoS ONE 8:e74832 10.1371/journal.pone.0074832 24023964 PMC3762779

[B262] Peet M. Murphy B. Shay J. Horrobin D. (1998). Depletion of omega-3 fatty acid levels in red blood cell membranes of depressive patients. Biol. Psychiatry 43, 315–319 10.1016/S0006-3223(97)00206-0 9513745

[B263] Pelleymounter M. A. Cullen M. J. Wellman C. L. (1995). Characteristics of BDNF-induced weight loss. Exp. Neurol. 131, 229–238 10.1016/0014-4886(95)90045-4 7534721

[B264] Pepino M. Y. Finkbeiner S. Mennella J. A. (2009). Similarities in food cravings and mood states between obese women and women who smoke tobacco. Obesity (Silver Spring) 17, 1158–1163 10.1038/oby.2009.46 19247281 PMC2757734

[B265] Perello M. Sakata I. Birnbaum S. Chuang J. C. Osborne-Lawrence S. Rovinsky S. A. (2010). Ghrelin increases the rewarding value of high-fat diet in an orexin-dependent manner. Biol. Psychiatry 67, 880–886 10.1016/j.biopsych.2009.10.030 20034618 PMC2854245

[B266] Perello M. Zigman J. M. (2012). The role of ghrelin in reward-based eating. Biol. Psychiatry 72, 347–353 10.1016/j.biopsych.2012.02.016 22458951 PMC3388148

[B267] Piazza P. L. M. Le Moal M. (1997). Glucocorticoids as a biological substrate of reward: physiological and pathophysiological implications. Brain Res. Rev. 25, 359–372 10.1016/S0165-0173(97)00025-8 9495563

[B268] Pickering C. Alsio J. Hulting A. L. Schioth H. B. (2009). Withdrawal from free-choice high-fat high-sugar diet induces craving only in obesity-prone animals. Psychopharmacology (Berl.) 204, 431–443 10.1007/s00213-009-1474-y 19205668

[B269] Pitchers K. K. Balfour M. E. Lehman M. N. Richtand N. M. Yu L. Coolen L. M. (2010). Neuroplasticity in the mesolimbic system induced by natural reward and subsequent reward abstinence. Biol. Psychiatry 67, 872–879 10.1016/j.biopsych.2009.09.036 20015481 PMC2854191

[B270] Pjetri E. Schmidt U. Kas M. J. Campbell I. C. (2012). Epigenetics and eating disorders. Curr. Opin. Clin. Nutr. Metab. Care 15, 330–335 10.1097/MCO.0b013e3283546fd3 22617563

[B271] Platt A. M. Egan A. M. Berquist M. J. Dreyer M. L. Babar G. Ugrasbul F. (2013). Health-related quality of life, depression, and metabolic parameters in overweight insulin-resistant adolescents. J. Pediatr. Health Care 27, 120–126 10.1016/j.pedhc.2011.06.015 23414977

[B272] Potenza M. N. (2014). Obesity, food, and addiction: emerging neuroscience and clinical and public health implications. Neuropsychopharmacology 39, 249–250 10.1038/npp.2013.198 24317324 PMC3857648

[B273] Prasad C. (1998). Food, mood and health: a neurobiologic outlook. Braz. J. Med. Biol. Res. 31, 1517–1527 10.1590/S0100-879X1998001200002 9951546

[B274] Pulkki-Raback L. Elovainio M. Kivimaki M. Mattsson N. Raitakari O. T. Puttonen S. (2009). Depressive symptoms and the metabolic syndrome in childhood and adulthood: a prospective cohort study. Health Psychol. 28, 108–116 10.1037/a0012646 19210024 PMC3166561

[B275] Rada P. Bocarsly M. E. Barson J. R. Hoebel B. G. Leibowitz S. F. (2010). Reduced accumbens dopamine in Sprague-Dawley rats prone to overeating a fat-rich diet. Physiol. Behav. 101, 394–400 10.1016/j.physbeh.2010.07.005 20643155 PMC2930885

[B276] Raison C. L. Miller A. H. (2003). When not enough is too much: the role of insufficient glucocorticoid signaling in the pathophysiology of stress-related disorders. Am. J. Psychiatry 160, 1554–1565 10.1176/appi.ajp.160.9.1554 12944327

[B277] Rangel A. (2013). Regulation of dietary choice by the decision-making circuitry. Nat. Neurosci. 16, 1717–1724 10.1038/nn.3561 24270272 PMC4053793

[B278] Reid M. Hammersley R. (1999). The effects of sucrose and maize oil on subsequent food intake and mood. Br. J. Nutr. 82, 447–455 10690160

[B279] Rho S. G. Kim Y. S. Choi S. C. Lee M. Y. (2014). Sweet food improves chronic stress-induced irritable bowel syndrome-like symptoms in rats. World J. Gastroenterol. 20, 2365–2373 10.3748/wjg.v20.i9.2365 24605034 PMC3942840

[B280] Richardson L. P. Davis R. Poulton R. Mccauley E. Moffitt T. E. Caspi A. (2003). A longitudinal evaluation of adolescent depression and adult obesity. Arch. Pediatr. Adolesc. Med. 157, 739–745 10.1001/archpedi.157.8.739 12912778

[B281] Roberts R. E. Deleger S. Strawbridge W. J. Kaplan G. A. (2003). Prospective association between obesity and depression: evidence from the Alameda County Study. Int. J. Obes. Relat. Metab. Disord. 27, 514–521 10.1038/sj.ijo.0802204 12664085

[B282] Robinson T. E. Berridge K. C. (2003). Addiction. Annu. Rev. Psychol. 54, 25–53 10.1146/annurev.psych.54.101601.145237 12185211

[B283] Rogers P. J. (1995). Food, mood and appetite. Nutr. Res. Rev. 8, 243–269 10.1079/NRR19950015 19094288

[B284] Rogers P. J. Lloyd H. M. (1994). Nutrition and mental performance. Proc. Nutr. Soc. 53, 443–456 10.1079/PNS19940049 7972158

[B285] Rolls B. J. Morris E. L. Roe L. S. (2002). Portion size of food affects energy intake in normal-weight and overweight men and women. Am. J. Clin. Nutr. 76, 1207–1213 12450884 10.1093/ajcn/76.6.1207

[B286] Romaguera D. Angquist L. Du H. Jakobsen M. U. Forouhi N. G. Halkjaer J. (2010). Dietary determinants of changes in waist circumference adjusted for body mass index–a proxy measure of visceral adiposity. PLoS ONE 5:e11588 10.1371/journal.pone.0011588 20644647 PMC2904387

[B287] Romaguera D. Norat T. Mouw T. May A. M. Bamia C. Slimani N. (2009). Adherence to the Mediterranean diet is associated with lower abdominal adiposity in European men and women. J. Nutr. 139, 1728–1737 10.3945/jn.109.108902 19571036

[B288] Rosenzweig-Lipson S. Sabb A. Stack G. Mitchell P. Lucki I. Malberg J. E. (2007). Antidepressant-like effects of the novel, selective, 5-HT2C receptor agonist WAY-163909 in rodents. Psychopharmacology (Berl.) 192, 159–170 10.1007/s00213-007-0710-6 17297636

[B289] Rossi S. De Chiara V. Musella A. Mataluni G. Sacchetti L. Siracusano A. (2010). Effects of caffeine on striatal neurotransmission: focus on cannabinoid CB1 receptors. Mol. Nutr. Food Res. 54, 525–531 10.1002/mnfr.200900237 20087854

[B290] Rubin R. T. Rhodes M. E. Czambel R. K. (2002). Sexual diergism of baseline plasma leptin and leptin suppression by arginine vasopressin in major depressives and matched controls. Psychiatry Res. 113, 255–268 10.1016/S0165-1781(02)00263-9 12559482

[B291] Ryan J. P. Sheu L. K. Critchley H. D. Gianaros P. J. (2012). A neural circuitry linking insulin resistance to depressed mood. Psychosom. Med. 74, 476–482 10.1097/PSY.0b013e31824d0865 22434915 PMC3372626

[B292] Ryo M. Nakamura T. Kihara S. Kumada M. Shibazaki S. Takahashi M. (2004). Adiponectin as a biomarker of the metabolic syndrome. Circ. J. 68, 975–981 10.1253/circj.68.975 15502375

[B293] Sadashiv Tiwari S. Paul B. N. Kumar S. Chandra A. Dhananjai S. (2012). Over expression of resistin in adipose tissue of the obese induces insulin resistance. World J. Diabetes 3, 135–141 10.4239/wjd.v3.i7.135 22816026 PMC3399912

[B294] Sampey B. P. Vanhoose A. M. Winfield H. M. Freemerman A. J. Muehlbauer M. J. Fueger P. T. (2011). Cafeteria diet is a robust model of human metabolic syndrome with liver and adipose inflammation: comparison to high-fat diet. Obesity (Silver Spring) 19, 1109–1117 10.1038/oby.2011.18 21331068 PMC3130193

[B295] Sanchez-Villegas A. Henriquez P. Figueiras A. Ortuno F. Lahortiga F. Martinez-Gonzalez M. A. (2007). Long chain omega-3 fatty acids intake, fish consumption and mental disorders in the SUN cohort study. Eur. J. Nutr. 46, 337–346 10.1007/s00394-007-0671-x 17717628

[B296] Sanchez-Villegas A. Martinez-Gonzalez M. A. (2013). Diet, a new target to prevent depression? BMC Med. 11:3 10.1186/1741-7015-11-3 23286788 PMC3570363

[B297] Sanchez-Villegas A. Martinez-Gonzalez M. A. Estruch R. Salas-Salvado J. Corella D. Covas M. I. (2013). Mediterranean dietary pattern and depression: the PREDIMED randomized trial. BMC Med. 11:208 10.1186/1741-7015-11-208 24229349 PMC3848350

[B298] Schachter S. (1968). Obesity and eating. Internal and external cues differentially affect the eating behavior of obese and normal subjects. Science 161, 751–756 10.1126/science.161.3843.751 5663800

[B299] Schaeffer M. Langlet F. Lafont C. Molino F. Hodson D. J. Roux T. (2013). Rapid sensing of circulating ghrelin by hypothalamic appetite-modifying neurons. Proc. Natl. Acad. Sci. U.S.A. 110, 1512–1517 10.1073/pnas.1212137110 23297228 PMC3557016

[B300] Schanze A. Reulbach U. Scheuchenzuber M. Groschl M. Kornhuber J. Kraus T. (2008). Ghrelin and eating disturbances in psychiatric disorders. Neuropsychobiology 57, 126–130 10.1159/000138915 18552514

[B301] Schellekens H. Clarke G. Jeffery I. B. Dinan T. G. Cryan J. F. (2012a). Dynamic 5-HT2C receptor editing in a mouse model of obesity. PLoS ONE 7:e32266 10.1371/journal.pone.0032266 22448217 PMC3308946

[B302] Schellekens H. Dinan T. G. Cryan J. F. (2013a). Ghrelin at the interface of obesity and reward. Vitam. Horm. 91, 285–323 10.1016/B978-0-12-407766-9.00013-4 23374722

[B303] Schellekens H. Dinan T. G. Cryan J. F. (2013b). Taking two to tango: a role for ghrelin receptor heterodimerization in stress and reward. Front. Neurosci. 7:148 10.3389/fnins.2013.00148 24009547 PMC3757321

[B304] Schellekens H. Finger B. C. Dinan T. G. Cryan J. F. (2012b). Ghrelin signalling and obesity: at the interface of stress, mood and food reward. Pharmacol. Ther. 10.1016/j.pharmthera.2012.06.004 22749794

[B305] Schellekens H. Van Oeffelen W. E. Dinan T. G. Cryan J. F. (2013c). Promiscuous dimerization of the growth hormone secretagogue receptor (GHS-R1a) attenuates ghrelin-mediated signaling. J. Biol. Chem. 288, 181–191 10.1074/jbc.M112.382473 23161547 PMC3537012

[B306] Schilling C. Gilles M. Blum W. F. Daseking E. Colla M. Weber-Hamann B. (2013). Leptin plasma concentrations increase during antidepressant treatment with amitriptyline and mirtazapine, but not paroxetine and venlafaxine: leptin resistance mediated by antihistaminergic activity? J. Clin. Psychopharmacol. 33, 99–103 10.1097/JCP.0b013e31827cb179 23277262

[B307] Schmauss C. (2003). Serotonin 2C receptors: suicide, serotonin, and runaway RNA editing. Neuroscientist 9, 237–242 10.1177/1073858403253669 12934707

[B308] Schultz W. (2000). Multiple reward signals in the brain. Nat. Rev. Neurosci. 1, 199–207 10.1038/35044563 11257908

[B309] Schultz W. (2002). Getting formal with dopamine and reward. Neuron 36, 241–263 10.1016/S0896-6273(02)00967-4 12383780

[B310] Schulze M. B. Fung T. T. Manson J. E. Willett W. C. Hu F. B. (2006). Dietary patterns and changes in body weight in women. Obesity (Silver Spring) 14, 1444–1453 10.1038/oby.2006.164 16988088

[B311] Schwartz D. R. Lazar M. A. (2011). Human resistin: found in translation from mouse to man. Trends Endocrinol. Metab. 22, 259–265 10.1016/j.tem.2011.03.005 21497511 PMC3130099

[B312] Schwartz G. J. (2000). The role of gastrointestinal vagal afferents in the control of food intake: current prospects. Nutrition 16, 866–873 10.1016/S0899-9007(00)00464-0 11054591

[B313] Shabbir F. Patel A. Mattison C. Bose S. Krishnamohan R. Sweeney E. (2013). Effect of diet on serotonergic neurotransmission in depression. Neurochem. Int. 62, 324–329 10.1016/j.neuint.2012.12.014 23306210

[B314] Sharma A. N. Elased K. M. Garrett T. L. Lucot J. B. (2010). Neurobehavioral deficits in db/db diabetic mice. Physiol. Behav. 101, 381–388 10.1016/j.physbeh.2010.07.002 20637218 PMC3098504

[B315] Sharma S. Fernandes M. F. Fulton S. (2013). Adaptations in brain reward circuitry underlie palatable food cravings and anxiety induced by high-fat diet withdrawal. Int. J. Obes. (Lond.) 37, 1183–1191 10.1038/ijo.2012.197 23229740

[B316] Sharma S. Fulton S. (2013). Diet-induced obesity promotes depressive-like behaviour that is associated with neural adaptations in brain reward circuitry. Int. J. Obes. (Lond.) 37, 382–389 10.1038/ijo.2012.48 22508336

[B317] Sharma S. Hryhorczuk C. Fulton S. (2012). Progressive-ratio responding for palatable high-fat and high-sugar food in mice. J. Vis. Exp. e3754 10.3791/3754 22588164 PMC3466943

[B318] Shen Q. Bergquist-Beringer S. (2013). Relationship between major depression and insulin resistance: does it vary by gender or race/ethnicity among young adults aged 20-39 years? J. Diabetes 5, 471–481 10.1111/1753-0407 23489875

[B319] Shinozaki G. Kumar Y. Rosen B. H. Rundell J. R. Mrazek D. A. Kung S. (2013). “Diminished” association between the serotonin transporter linked polymorphism (5HTTLPR) and body mass index in a large psychiatric sample. J. Affect. Disord. 151, 397–400 10.1016/j.jad.2013.06.021 23838390

[B320] Silberberg G. Lundin D. Navon R. Ohman M. (2012). Deregulation of the A-to-I RNA editing mechanism in psychiatric disorders. Hum. Mol. Genet. 21, 311–321 10.1093/hmg/ddr461 21984433

[B321] Silha J. V. Krsek M. Skrha J. Sucharda P. Nyomba B. L. Murphy L. J. (2004). Plasma resistin, leptin and adiponectin levels in non-diabetic and diabetic obese subjects. Diabet. Med. 21, 497–499 10.1111/j.1464-5491.2004.01178.x 15089799

[B322] Simon G. E. Von Korff M. (2006). Medical co-morbidity and validity of DSM-IV depression criteria. Psychol. Med. 36, 27–36 10.1017/S0033291705006136 16202189

[B323] Simon G. E. Von Korff M. Saunders K. Miglioretti D. L. Crane P. K. Van Belle G. (2006). Association between obesity and psychiatric disorders in the US adult population. Arch. Gen. Psychiatry 63, 824–830 10.1001/archpsyc.63.7.824 16818872 PMC1913935

[B324] Singh M. Kesterson R. A. Jacobs M. M. Joers J. M. Gore J. C. Emeson R. B. (2007). Hyperphagia-mediated obesity in transgenic mice misexpressing the RNA-editing enzyme ADAR2. J. Biol. Chem. 282, 22448–22459 10.1074/jbc.M700265200 17567573

[B325] Singh M. Singh M. M. Na E. Agassandian K. Zimmerman M. B. Johnson A. K. (2011). Altered ADAR 2 equilibrium and 5HT(2C) R editing in the prefrontal cortex of ADAR 2 transgenic mice. Genes Brain Behav. 10, 637–647 10.1111/j.1601-183X.2011.00701.x 21615684 PMC3150620

[B326] Singh M. Zimmerman M. B. Beltz T. G. Johnson A. K. (2009). Affect-related behaviors in mice misexpressing the RNA editing enzyme ADAR2. Physiol. Behav. 97, 446–454 10.1016/j.physbeh.2009.03.029 19361536 PMC2778280

[B327] Sinha R. Jastreboff A. M. (2013). Stress as a common risk factor for obesity and addiction. Biol. Psychiatry 73, 827–835 10.1016/j.biopsych.2013.01.032 23541000 PMC3658316

[B328] Siuciak J. A. Altar C. A. Wiegand S. J. Lindsay R. M. (1994). Antinociceptive effect of brain-derived neurotrophic factor and neurotrophin-3. Brain Res. 633, 326–330 10.1016/0006-8993(94)91556-3 7511037

[B329] Siuciak J. A. Lewis D. R. Wiegand S. J. Lindsay R. M. (1997). Antidepressant-like effect of brain-derived neurotrophic factor (BDNF). Pharmacol. Biochem. Behav. 56, 131–137 10.1016/S0091-3057(96)00169-4 8981620

[B330] Skibicka K. P. Dickson S. L. (2011). Ghrelin and food reward: the story of potential underlying substrates. Peptides 32, 2265–2273 10.1016/j.peptides.2011.05.016 21621573

[B331] Skibicka K. P. Hansson C. Alvarez-Crespo M. Friberg P. A. Dickson S. L. (2011). Ghrelin directly targets the ventral tegmental area to increase food motivation. Neuroscience 180, 129–137 10.1016/j.neuroscience.2011.02.016 21335062

[B332] Sodhi M. S. Burnet P. W. Makoff A. J. Kerwin R. W. Harrison P. J. (2001). RNA editing of the 5-HT(2C) receptor is reduced in schizophrenia. Mol. Psychiatry 6, 373–379 10.1038/sj.mp.4000920 11443520

[B333] Solomon M. B. Furay A. R. Jones K. Packard A. E. Packard B. A. Wulsin A. C. (2012). Deletion of forebrain glucocorticoid receptors impairs neuroendocrine stress responses and induces depression-like behavior in males but not females. Neuroscience 203, 135–143 10.1016/j.neuroscience.2011.12.014 22206943 PMC4000316

[B334] Souquet A. M. Rowland N. E. (1989). Effect of chronic administration of dexfenfluramine on stress- and palatability-induced food intake in rats. Physiol. Behav. 46, 145–149 10.1016/0031-9384(89)90247-3 2602451

[B335] Spence S. Courbasson C. (2012). The role of emotional dysregulation in concurrent eating disorders and substance use disorders. Eat. Behav. 13, 382–385 10.1016/j.eatbeh.2012.05.006 23121793

[B336] Spring B. M. O. Wurtman J. Digman L. Cozolino L. (1982-1983). Effects of protein and carbohydrate meals on mood and performance: interactions with sex and age. J. Psychiatry Res. 17, 155–167 6764932 10.1016/0022-3956(82)90017-6

[B337] Stahl L. A. Begg D. P. Weisinger R. S. Sinclair A. J. (2008). The role of omega-3 fatty acids in mood disorders. Curr. Opin. Investig. Drugs 9, 57–64 18183532

[B338] Steiger H. (2004). Eating disorders and the serotonin connection: state, trait and developmental effects. J. Psychiatry Neurosci. 29, 20–29 14719047 PMC305267

[B339] Steppan C. M. Bailey S. T. Bhat S. Brown E. J. Banerjee R. R. Wright C. M. (2001). The hormone resistin links obesity to diabetes. Nature 409, 307–312 10.1038/35053000 11201732

[B340] Stetler C. Miller G. E. (2011). Depression and hypothalamic-pituitary-adrenal activation: a quantitative summary of four decades of research. Psychosom. Med. 73, 114–126 10.1097/PSY.0b013e31820ad12b 21257974

[B341] Stice E. Dagher A. (2010). Genetic variation in dopaminergic reward in humans. Forum Nutr. 63, 176–185 10.1159/000264405 19955785

[B342] Stice E. Yokum S. Bohon C. Marti N. Smolen A. (2010). Reward circuitry responsivity to food predicts future increases in body mass: moderating effects of DRD2 and DRD4. Neuroimage 50, 1618–1625 10.1016/j.neuroimage.2010.01.081 20116437 PMC3987805

[B343] Suzuki K. Jayasena C. N. Bloom S. R. (2012). Obesity and appetite control. Exp. Diabetes Res. 2012:824305 10.1155/2012/824305 22899902 PMC3415214

[B344] Suzuki K. Simpson K. A. Minnion J. S. Shillito J. C. Bloom S. R. (2010). The role of gut hormones and the hypothalamus in appetite regulation. Endocr. J. 57, 359–372 10.1507/endocrj.K10E-077 20424341

[B345] Talen M. R. Mann M. M. (2009). Obesity and mental health. Prim. Care 36, 287–305 10.1016/j.pop.2009.01.012 19501244

[B346] Taylor V. H. Curtis C. M. Davis C. (2010). The obesity epidemic: the role of addiction. CMAJ 182, 327–328 10.1503/cmaj.091142 20026623 PMC2831667

[B347] Tchernof A. Despres J. P. (2013). Pathophysiology of human visceral obesity: an update. Physiol. Rev. 93, 359–404 10.1152/physrev.00033.2011 23303913

[B348] Teegarden S. L. Bale T. L. (2007). Decreases in dietary preference produce increased emotionality and risk for dietary relapse. Biol. Psychiatry 61, 1021–1029 10.1016/j.biopsych.2006.09.032 17207778

[B349] Teegarden S. L. Bale T. L. (2008). Effects of stress on dietary preference and intake are dependent on access and stress sensitivity. Physiol. Behav. 93, 713–723 10.1016/j.physbeh.2007.11.030 18155095 PMC2483328

[B350] Tryon M. S. Carter C. S. Decant R. Laugero K. D. (2013). Chronic stress exposure may affect the brain's response to high calorie food cues and predispose to obesogenic eating habits. Physiol. Behav. 120, 233–242 10.1016/j.physbeh.2013.08.010 23954410

[B351] Tschop M. Smiley D. L. Heiman M. L. (2000). Ghrelin induces adiposity in rodents. Nature 407, 908–913 10.1038/35038090 11057670

[B352] Tsuboi H. Watanabe M. Kobayashi F. Kimura K. Kinae N. (2013). Associations of depressive symptoms with serum proportions of palmitic and arachidonic acids, and alpha-tocopherol effects among male population–a preliminary study. Clin. Nutr. 32, 289–293 10.1016/j.clnu.2012.07.011 22901744

[B353] Turer A. T. Scherer P. E. (2012). Adiponectin: mechanistic insights and clinical implications. Diabetologia 55, 2319–2326 10.1007/s00125-012-2598-x 22688349

[B354] Tzschentke T. M. (2001). Pharmacology and behavioral pharmacology of the mesocortical dopamine system. Prog Neurobiol 63, 241–320 10.1016/S0301-0082(00)00033-2 11115727

[B355] Ulrich-Lai Y. M. Christiansen A. M. Ostrander M. M. Jones A. A. Jones K. R. Choi D. C. (2010). Pleasurable behaviors reduce stress via brain reward pathways. Proc. Natl. Acad. Sci. U.S.A. 107, 20529–20534 10.1073/pnas.1007740107 21059919 PMC2996660

[B356] Ulrich-Lai Y. M. Herman J. P. (2009). Neural regulation of endocrine and autonomic stress responses. Nat. Rev. Neurosci. 10, 397–409 10.1038/nrn2647 19469025 PMC4240627

[B357] Van Reedt Dortland A. K. Giltay E. J. Van Veen T. Zitman F. G. Penninx B. W. (2013a). Longitudinal relationship of depressive and anxiety symptoms with dyslipidemia and abdominal obesity. Psychosom. Med. 75, 83–89 10.1097/PSY.0b013e318274d30f 23197842

[B358] Van Reedt Dortland A. K. Vreeburg S. A. Giltay E. J. Licht C. M. Vogelzangs N. Van Veen T. (2013b). The impact of stress systems and lifestyle on dyslipidemia and obesity in anxiety and depression. Psychoneuroendocrinology 38, 209–218 10.1016/j.psyneuen.2012.05.01 22717171

[B359] Van Strater A. C. Bouvy P. F. (2006). Omega-3 fatty acids and mood disorders. Am. J. Psychiatry 163:2018 10.1176/appi.ajp.163.11.2018 17074963

[B360] Volkow N. D. O'brien C. P. (2007). Issues for DSM-V: should obesity be included as a brain disorder? Am. J. Psychiatry 164, 708–710 10.1176/appi.ajp.164.5.708 17475727

[B361] Volkow N. D. Wang G. J. Baler R. D. (2011). Reward, dopamine and the control of food intake: implications for obesity. Trends Cogn. Sci. 15, 37–46 10.1016/j.tics.2010.11.001 21109477 PMC3124340

[B362] Volkow N. D. Wang G. J. Fowler J. S. Tomasi D. Baler R. (2012). Food and drug reward: overlapping circuits in human obesity and addiction. Curr. Top. Behav. Neurosci. 11, 1–24 10.1007/7854_2011_169 22016109

[B363] Volkow N. D. Wang G. J. Fowler J. S. Tomasi D. Telang F. Baler R. (2010). Addiction: decreased reward sensitivity and increased expectation sensitivity conspire to overwhelm the brain's control circuit. Bioessays 32, 748–755 10.1002/bies.201000042 20730946 PMC2948245

[B364] Volkow N. D. Wang G. J. Tomasi D. Baler R. D. (2013). Obesity and addiction: neurobiological overlaps. Obes. Rev. 14, 2–18 10.1111/j.1467-789X.2012.01031.x 23016694 PMC4827343

[B365] Wallin M. S. Rissanen A. M. (1994). Food and mood: relationship between food, serotonin and affective disorders. Acta Psychiatry Scand. Suppl. 377, 36–40 10.1111/j.1600-0447.1994.tb05800.x 8053364

[B366] Wang G. J. Volkow N. D. Telang F. Jayne M. Ma J. Rao M. (2004a). Exposure to appetitive food stimuli markedly activates the human brain. Neuroimage 21, 1790–1797 10.1016/j.neuroimage.2003.11.026 15050599

[B367] Wang G. J. Volkow N. D. Thanos P. K. Fowler J. S. (2004b). Similarity between obesity and drug addiction as assessed by neurofunctional imaging: a concept review. J. Addict. Dis. 23, 39–53 10.1300/J069v23n03_04 15256343

[B368] Wang G. J. Volkow N. D. Thanos P. K. Fowler J. S. (2009). Imaging of brain dopamine pathways: implications for understanding obesity. J. Addict. Med. 3, 8–18 10.1097/ADM.0b013e31819a86f7 21603099 PMC3098897

[B369] Wang Q. Verweij E. W. Krugers H. J. Joels M. Swaab D. F. Lucassen P. J. (2013). Distribution of the glucocorticoid receptor in the human amygdala; changes in mood disorder patients. Brain Struct. Funct. [Epub ahead of print]. 10.1007/s00429-013-0589-4 23748930

[B370] Way J. M. Gorgun C. Z. Tong Q. Uysal K. T. Brown K. K. Harrington W. W. (2001). Adipose tissue resistin expression is severely suppressed in obesity and stimulated by peroxisome proliferator-activated receptor gamma agonists. J. Biol. Chem. 276, 25651–25653 10.1074/jbc.C100189200 11373275

[B371] Weatherford S. C. Greenberg D. Gibbs J. Smith G. P. (1990). The potency of D-1 and D-2 receptor antagonists is inversely related to the reward value of sham-fed corn oil and sucrose in rats. Pharmacol. Biochem. Behav. 37, 317–323 10.1016/0091-3057(90)90341-E 2150443

[B372] Weber-Hamann B. Hentschel F. Kniest A. Deuschle M. Colla M. Lederbogen F. (2002). Hypercortisolemic depression is associated with increased intra-abdominal fat. Psychosom. Med. 64, 274–277 10.1097/00006842-200203000-00010 11914443

[B373] Weber-Hamann B. Kratzsch J. Kopf D. Lederbogen F. Gilles M. Heuser I. (2007). Resistin and adiponectin in major depression: the association with free cortisol and effects of antidepressant treatment. J. Psychiatry Res. 41, 344–350 10.1016/j.jpsychires.2006.01.002 16497334

[B374] Wei Q. Lu X. Y. Liu L. Schafer G. Shieh K. R. Burke S. (2004). Glucocorticoid receptor overexpression in forebrain: a mouse model of increased emotional lability. Proc. Natl. Acad. Sci. U.S.A. 101, 11851–11856 10.1073/pnas.0402208101 15280545 PMC511063

[B375] Weltens N. Zhao D. Van Oudenhove L. (2014). Where is the comfort in comfort foods? Mechanisms linking fat signaling, reward, and emotion. Neurogastroenterol. Motil. 26, 303–315 10.1111/nmo.12309 24548257

[B376] Westling S. Ahren B. Traskman-Bendz L. Westrin A. (2004). Low CSF leptin in female suicide attempters with major depression. J. Affect. Disord. 81, 41–48 10.1016/j.jad.2003.07.002 15183598

[B377] Wilhelm C. J. Choi D. Huckans M. Manthe L. Loftis J. M. (2013). Adipocytokine signaling is altered in Flinders sensitive line rats, and adiponectin correlates in humans with some symptoms of depression. Pharmacol. Biochem. Behav. 103, 643–651 10.1016/j.pbb.2012.11.001 23153628 PMC4408933

[B378] Williams J. Mobarhan S. (2003). A critical interaction: leptin and ghrelin. Nutr. Rev. 61, 391–393 10.1301/nr.2003.nov.391-393 14677575

[B379] Wurtman J. J. (1993). Depression and weight gain: the serotonin connection. J. Affect. Disord. 29, 183–192 10.1016/0165-0327(93)90032-F 8300977

[B380] Wurtman R. J. Wurtman J. J. (1989). Carbohydrates and depression. Sci. Am. 260, 68–75 10.1038/scientificamerican0189-68 2642626

[B381] Wurtman R. J. Wurtman J. J. (1996). Brain Serotonin, Carbohydrate-craving, obesity and depression. Adv. Exp. Med. Biol. 398, 35–41 10.1007/978-1-4613-0381-7_4 9045545

[B382] Yamada N. Katsuura G. Ochi Y. Ebihara K. Kusakabe T. Hosoda K. (2011). Impaired CNS leptin action is implicated in depression associated with obesity. Endocrinology 152, 2634–2643 10.1210/en.2011-0004 21521746

[B383] Yau Y. H. Potenza M. N. (2013). Stress and eating behaviors. Minerva Endocrinol. 38, 255–267 24126546 PMC4214609

[B384] Ye J. Gao Z. Yin J. He Q. (2007). Hypoxia is a potential risk factor for chronic inflammation and adiponectin reduction in adipose tissue of ob/ob and dietary obese mice. Am. J. Physiol. Endocrinol. Metab. 293, E1118–E1128 10.1152/ajpendo.00435.2007 17666485

[B385] Yilmaz Y. (2008). Psychopathology in the context of obesity: the adiponectin hypothesis. Med. Hypotheses 70, 902–903 10.1016/j.mehy.2007.08.019 17920209

[B386] Young C. Martin A. (2003). Omega-3 fatty acids in mood disorders: an overview. Rev. Bras. Psiquiatr. 25, 184–187 10.1590/S1516-44462003000300012 12975694

[B387] Young S. N. (2007). Folate and depression–a neglected problem. J. Psychiatry Neurosci. 32, 80–82 17353937 PMC1810582

[B388] Zagon A. (2001). Does the vagus nerve mediate the sixth sense? Trends Neurosci. 24, 671–673 10.1016/S0166-2236(00)01929-9 11672813

[B389] Zeman M. Jirak R. Jachymova M. Vecka M. Tvrzicka E. Zak A. (2009). Leptin, adiponectin, leptin to adiponectin ratio and insulin resistance in depressive women. Neuro Endocrinol. Lett. 30, 387–395 19855365

[B390] Zeng B. Y. Heales S. J. Canevari L. Rose S. Jenner P. (2004). Alterations in expression of dopamine receptors and neuropeptides in the striatum of GTP cyclohydrolase-deficient mice. Exp. Neurol. 190, 515–524 10.1016/j.expneurol.2004.08.022 15530890

[B391] Zhao G. Ford E. S. Li C. Tsai J. Dhingra S. Balluz L. S. (2011). Waist circumference, abdominal obesity, and depression among overweight and obese U.S. adults: National Health and Nutrition Examination Survey 2005-2006. BMC Psychiatry 11:130 10.1186/1471-244X-11-130 21834955 PMC3163524

[B392] Ziauddeen H. Farooqi I. S. Fletcher P. C. (2012). Obesity and the brain: how convincing is the addiction model? Nat. Rev. Neurosci. 13, 279–286 10.1038/nrn3212 22414944

[B393] Ziauddeen H. Fletcher P. C. (2013). Is food addiction a valid and useful concept? Obes. Rev. 14, 19–28 10.1111/j.1467-789X.2012.01046.x 23057499 PMC3561707

